# TAOK2 controls synaptic plasticity and anxiety via ERK and calcium signaling

**DOI:** 10.1016/j.isci.2025.113712

**Published:** 2025-10-09

**Authors:** Wenbo Ma, Inanna Warnhoff, Marius Stephan, Xiao Ma, Kerstin Dehne, Paul Volkmann, Nirmal Kannaiyan, Ben Brankatschk, Niels Jensen, Moritz J. Rossner, Volker Scheuss, Michael C. Wehr

**Affiliations:** 1 Research Group Cell Signalling, Department of Psychiatry and Psychotherapy, LMU University Hospital, LMU Munich, Nussbaumstr. 7, 80336 Munich, Germany; 2 Research Group Neurophysiology of Psychiatric Disease, Department of Psychiatry and Psychotherapy, LMU University Hospital, LMU Munich, Nussbaumstr. 7, 80336 Munich, Germany; 3 Systasy Bioscience GmbH, Fraunhoferstr. 8, 82152 Planegg, Germany; 4 Molecular Neurobiology, Department of Psychiatry and Psychotherapy, LMU University Hospital, LMU Munich, Nussbaumstr. 7, 80336 Munich, Germany; 5 Centre for Neural Circuits and Behaviour, University of Oxford, Oxford OX1 3SR, UK; 6 Department of Animal Physiology, RPTU University Kaiserslautern-Landau, Kaiserslautern, Germany

**Keywords:** Molecular neuroscience, Cellular neuroscience, Transcriptomics

## Abstract

The kinase thousand and one amino acid kinase 2 (TAOK2) regulates dendritic architecture and synaptic plasticity and is implicated in neurodevelopmental and neuropsychiatric disorders, including autism and schizophrenia. Here, we investigated TAOK2 function by creating an *Emx1-Cre*-driven, excitatory-neuron-specific conditional *Taok2* knockout (*Taok2* cKO) mouse line. Pathway profiling in *Taok2* cKO primary cortical neurons revealed impaired extracellular regulated kinase (ERK)/mitogen-activated protein kinase (MAPK) and calcium signaling after AMPA, BDNF, or bicuculline stimulation. These results were validated by reduced p-ERK1/2 protein levels and decreased calcium flux. Cultured *Taok2* cKO neurons displayed reduced synaptic density and connectivity. Single-nucleus RNA sequencing of medial prefrontal cortex identified dysregulated gene expression enriched for postsynaptic MAPK and calcium pathways within cortical layers 2/3 and 4/5. *Taok2* cKO mice exhibited an anxiety-related thigmotactic behavior in the open field test. Our findings demonstrate that TAOK2 loss in excitatory cortical neurons disrupts synaptic signaling and connectivity, drives behavioral abnormalities, and positions TAOK2 as a potential drug target for neuropsychiatric disorders.

## Highlights

• TAOK2 loss in excitatory cortical neurons disrupts ERK/MAPK and calcium signaling
• TAOK2-deficient neurons show reduced synaptic density and connectivity in culture
• snRNA-seq reveals transcriptional changes in cortical postsynaptic signaling
• *Taok2* conditional knockout mice display anxiety-related behavior in open field test

## Graphical abstract

**Figure:**
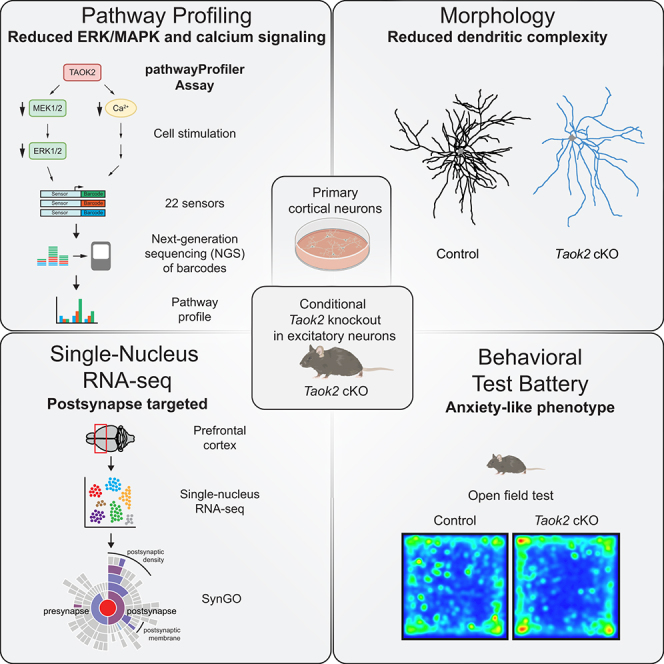

## Introduction

Thousand and one amino acid kinase 2 (TAOK2) is a serine/threonine kinase of the Ste20-like family and is located in humans at the chromosomal locus 16p11.2.^1^ Clinically, TAOK2 is associated with the risk for mental and neurodevelopmental disorders.^2^^,^^3^^,^^4^ A heterozygous deletion of the 16p11.2 locus, which contains 27 protein coding genes, including TAOK2, is linked to autism spectrum disorders,^5^ while a duplication of the same chromosomal region is associated with bipolar disorder, autism spectrum disorder (ASD), and schizophrenia.^2^^,^^5^^,^^6^ Also, SNPs located in the TAOK2 locus confer genetic susceptibility to autism and schizophrenia.^3^^,^^7^ Recently, patients with TAOK2 variants were reported to exhibit neurodevelopmental abnormalities, with 75% showing macrocephaly and autism phenotypes, further highlighting the clinical relevance of TAOK2 variants.^4^ TAOK2 is also associated with neurodegenerative disorders such as Alzheimer disease, as it phosphorylates tau protein, which forms pathogenic fibrils when hyperphosphorylated.^8^^,^^9^ In mice, full inactivation of *Taok2* in the forebrain causes a hyperactivity phenotype^10^ as well as deficits in cognition, anxiety, and social interaction.^11^ At the molecular and morphological levels, TAOK2 regulates actin dynamics and promotes arborization of neurons.^12^ Phosphorylation of TAOK2 by the serine-threonine kinase 24 (STK24) regulates the development of synapses via interaction with myosin Va.^13^ In addition, TAOK2 also promotes the formation of mature spines by associating with septin 7, which in turn stabilizes PSD-95, a key scaffold of the postsynaptic density network.^14^ Furthermore, TAOK2 activates mitogen-activated protein kinases (MAPKs) in neurons, including c-Jun N-terminal kinase (JNK) and p38, and regulates synaptic plasticity through RhoA signaling.^15^^,^^16^ TAOK2 also activates Hippo signaling, a pathway that controls cell fate decision, cell polarity maintenance, and actin cytoskeleton dynamics in neurons.^17^ Notably, two *de novo* mutations, A135P and P1022∗, were recently found in ASD families to affect the kinase activity of TAOK2 and the development of neuronal spines and caused aberrant activity of c-Jun N-terminal kinase (JNK).^18^ Conversely, inactivation of TAOK2 leads to mislocalization of synapses to dendritic shafts and defects in calcium compartmentalization.^19^

TAOK2 is known to modulate synaptic plasticity, the ability of synapses to strengthen and weaken over time in response to changes in activity. Synaptic plasticity is important for neuronal development, learning and memory processes, adaptation, and underlying pathologies.^20^ Cellular signaling cascades that are initiated in dendritic spines induce post-translational modifications of signaling proteins and finally signal to the nucleus to regulate gene expression, thus integrating changes of synaptic plasticity. For example, the regulated influx of calcium ions at spines initiates calcium-regulated signaling pathways.^21^ Furthermore, activated MAPK pathways that lead to elevated phosphorylation levels of MAPKs, such as the extracellular regulated kinases (ERK1/2), JNK, and p38, as well as cytoskeleton rearrangements mediated through RhoA signaling have been linked to the modulation of synaptic plasticity.^22^ Among these, the ERK1/2 pathway plays a central role in mediating long-term synaptic strengthening by regulating AMPA receptor trafficking, synaptogenesis, and gene expression. The pharmacological increase of ERK activity has been shown to amplify and prolong activity-dependent synaptic potentiation in the hippocampus.^23^

In this study, we used (1) a multiplexed cell-based pathway profiling assay using barcoded pathway biosensors, (2) morphological studies to evaluate the impact of *Taok2* inactivation on cellular signaling and synaptic plasticity, (3) single-nucleus RNA sequencing (snRNA-seq) of prefrontal cortex tissue of mice in which floxed *Taok2* alleles were selectively inactivated in excitatory neurons by an *Emx1*-driven Cre recombinase, and (4) a comprehensive neurocognitive behavioral test battery in conditional *Taok2* mutant mice to assess the impact of a specific *Taok2* inactivation in excitatory neurons. Using both cell-based and *in vivo* approaches, we demonstrate that Taok2-dependent dysregulation of ERK/MAPK and calcium signaling impairs synaptic plasticity and causes behavioral abnormalities.

## Results

### A conditional Taok2 knockout mouse line for cell-based assays and behavioral profiling

Previously, it was reported that constitutive homozygous *Taok2* knockout mice showed behavioral abnormalities in cognition, anxiety, and social interaction.^24^ We aimed to investigate if a conditional inactivation of *Taok2* selectively in excitatory neurons still results in altered behavioral phenotypes and to characterize this model at the molecular and morphological levels in neuron cultures. Therefore, we generated floxed *Taok2* mice from a commercially available embryonal stem cell clone purchased from EUCOMM. Cre-recombinase-mediated recombination of *loxP* sites flanking exons 4 to 8 of the *Taok2* gene results in a premature stop codon and leads to a functional deletion of *Taok2* (Figures S1A–S1D). We used an *Emx1*-promoter-driven *Cre* recombinase driver line where expression of *Cre* starts as early as embryonic day 9.5 in the brain to specifically inactivate *Taok2* expression in excitatory neurons.^25^ The genetic inactivation of *Taok2* in cortical excitatory neurons was validated by western blotting for Taok2 protein (Figure S1E).

### Pathway activity profiling reveals reduced activity in MAPK and calcium signaling in neuron culture

**Figure 1:**
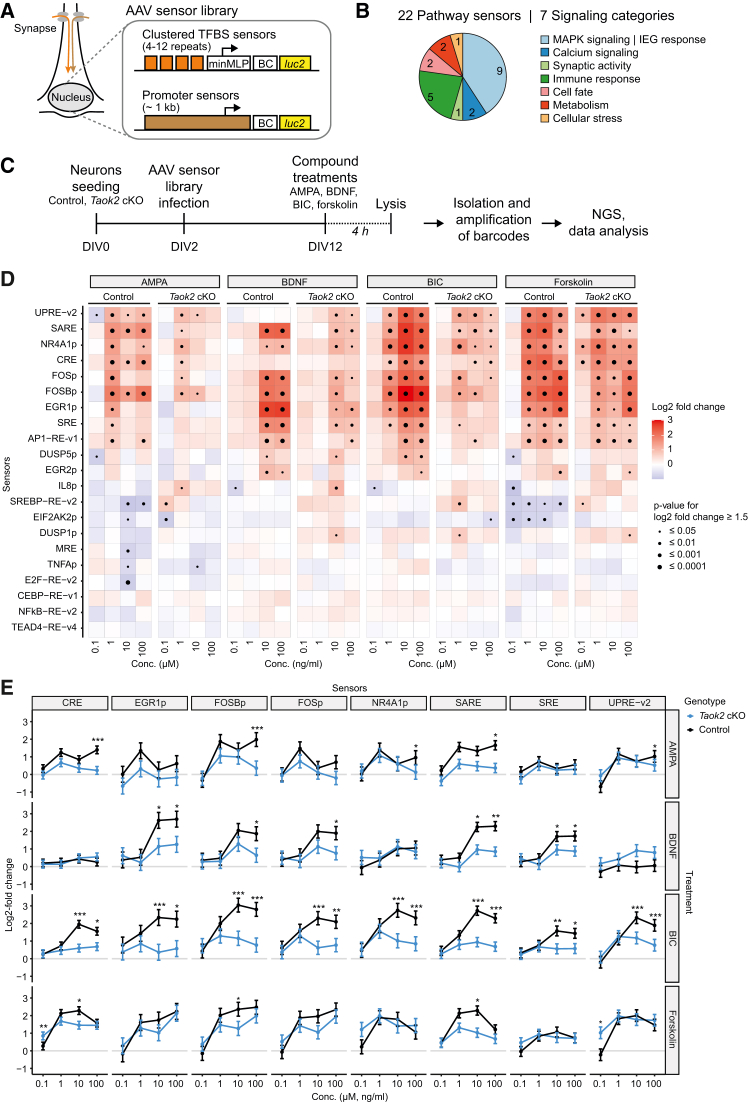
Pathway profiling indicated reduced MAPK and calcium signaling in *Taok2* cKO neurons (A) Schematic principle of the barcoded pathwayProfiler assay in neurons. Pathway reporters consist of either clustered transcription factor binding sites (TFBS) fused to a minimal major late promoter (minMLP) or human promoters. Each sensor is linked to an RNA barcode (BC) reporter and a luciferase (*luc2*) reporter gene. AAV, adeno-associated virus. (B) Pie chart showing the distribution of pathway sensors by signaling category. The AAV sensor library contained a total of 22 sensors. Numbers inside the pie chart indicate the number of sensors for each category. (C) Experimental outline for the pathwayProfiler assay in mouse primary neurons. BDNF, brain-derived neurotrophic factor; BIC, bicuculline; NGS, next-generation sequencing. (D) Heatmap of 4-h stimulation response of control and *Taok2* cKO primary cortical neurons to AMPA, BDNF, BIC, and forskolin. Primary neurons were stimulated on day 12 *in vitro* for 4 h. *n* = 4. ∗*p* ≤ 0.05; ∗∗*p* ≤ 0.01; ∗∗∗*p* ≤ 0.001; ∗∗∗∗*p* ≤ 0.0001, and Wald test with Benjamini-Hochberg (BH) correction. For data points with an absolute log2 fold change greater than 1.5, a significance value was calculated. (E) Selected concentration-dependent sensor responses to AMPA, BDNF, BIC, and forskolin treatment visualizing the genetic effect of *Taok2* deficiency. Lines represent means, error bars represent SEM; *n* = 4. Same statistics as in (D). See also Figure S2 and Table S1.

To study the role of TAOK2 at the molecular level in neurons, we used the “pathwayProfiler” assay (Systasy Bioscience), a multiparametric-cell-based pathway profiling assay platform that can monitor activities of multiple signaling pathways in cultured neurons in parallel.^26^ The assay uses genetically encoded RNA barcode reporters coupled to performance-optimized pathway sensors to profile signaling activities in living cells. The transcribed barcodes, driven by an activated sensor, are analyzed by next-generation sequencing (NGS) and allow the simultaneous measurement of multiple individual signaling events from a single well in parallel. The sensors consist of either synthetic repetitive DNA motifs that recruit single or a small number of transcription factors or native human promoter sequences that usually recruit a wide range of different transcription factors (Figure 1A). These nuclear sensors function as the distal endpoints of the cellular signaling pathways. The pathways covered by the “pathwayProfiler” include various signaling cascades related to synaptic activity and calcium signaling, MAPK signaling and immediate-early gene (IEG) response, cell fate, regulation of cellular stress, immune response, metabolism, and stem cell pluripotency (Figure 1B; Table S1). The pathwayProfiler assay was performed in primary neurons isolated from the cortex of *Taok2 (fl/fl) x Emx1-Cre (tg/0)* knockout mice (*Taok2* cKO neurons) and littermate controls at embryonic day E15.5. To analyze the impact of *Taok2* inactivation on synapse signaling, we treated *Taok2* cKO neurons and control neurons with increasing concentrations of AMPA (agonist of the AMPA receptor), brain-derived neurotrophic factor (BDNF; agonist of the TrkB receptor), bicuculline (BIC; blocks inhibitory GABA-A receptors), and forskolin (cAMP agonist, used as a technical positive control) for 4 h on day 12 of *in vitro* differentiation (DIV 12) (Figure 1C). All four stimulations resulted in a characteristic pattern of neuronal-activity-dependent activation of sensors (Figure 1D). AMPA, BIC, and forskolin stimulated both MAPK-specific sensors (e.g., EGR1p, FOSp, FOSBp, NR4A1p, SRE, and AP1-RE-v1) and calcium-responsive sensors (UPRE-v2 and CRE),^26^ whereas BDNF exclusively activated the MAPK sensors. Notably, BDNF treatment did not activate any of the calcium-responsive sensors. This supports the notion that BDNF acts as a selective stimulator of MAPK signaling.^27^ When comparing the stimulated sensors in control vs. *Taok2* cKO neurons, we found that treatment with AMPA, BDNF, and BIC, but not the forskolin control, resulted in significantly reduced activities of several sensors in mutant neurons (Figure S2A). The pronounced genotype effect was seen for the neuronal-activity-dependent MAPK sensors (EGR1p, FOSp, FOSBp, and SRE), the calcium sensors (CRE, UPRE-v2, and NR4A1p), and the synaptic activity response element (SARE) sensor. The latter contains binding sites for the transcription factors CREB, MEF2, and SRF/TCF and can capture both MAPK and calcium signals.^28^ The other, more specific sensors for MAPK signaling and IEG response are largely based on the promoter sequences of neuronal-activity-induced IEGs, such as EGR1, FOS, or FOSB, but also include synthetic reporters, such as SRE. Notably, AMPA and BDNF treatments also resulted in robustly reduced responses of CRE, EGR1p, FOSBp, FOSp, SARE, SRE, and UPRE-v2 sensors in *Taok2* cKO neurons (Figure 1E). However, BIC treatment caused the greatest reduction in sensor responses compared to controls. To assess whether these stimulation-induced changes were sustained, sensor responses were also measured 24 h after treatment (Figure S2B). Sensor responses at this later time point were now similar for both control and *Taok2* cKO neurons, arguing that all stimuli, except forskolin, have only a transient effect in these cells (Figure S2C). Taken together, *Taok2* inactivation in excitatory neurons reduced the activity of several sensors that respond to synaptic activity through both MAPK and calcium signaling.

### TAOK2 regulates synaptic ERK and calcium signaling

**Figure 2:**
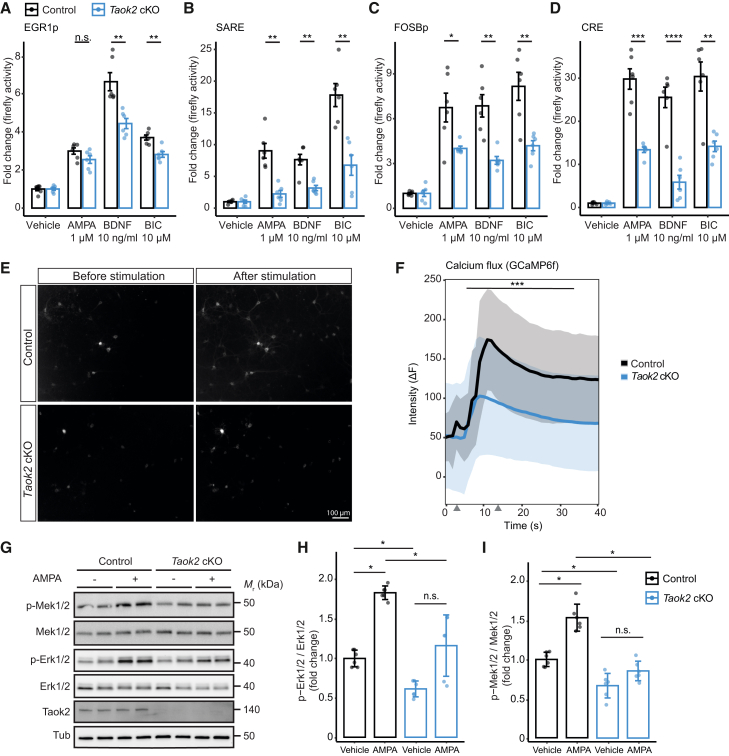
MAPK and calcium signaling is reduced in *Taok2* cKO neurons (A–D) Luciferase assay using EGR1p (A), SARE- (B), FOSBp- (C), and CRE- (D) sensor-detected MAPK (A–C) and calcium (B, D)-dependent signaling activity in primary cortical neurons. Neurons were treated with AMPA (1 μM), BDNF (10 ng/mL), or bicuculline (BIC, 1 μM) on day *in vitro* 12 (DIV12) for 4 h. ∗*p* < 0.05; ∗∗*p* < 0.01; ∗∗∗*p* < 0.001; ∗∗∗∗*p* < 0.0001; n.s., not significant; Student’s t test (two-sided). (E) Imaging of control and *Taok2* cKO primary cortical neurons infected with AVVs expressing the synapsin1-driven GCaMP6f calcium sensor. Images were taken before and after stimulating cells with 25 mM KCl. (F) Quantification of cellular GCaMP6f responses to 25 mM KCl. Intensities from responding cells were normalized to the background and pooled for analysis. Gray arrowheads on the *x* axis represent the time points at which images were taken before and after stimulation as shown in (E). The line represents the mean, and the shaded area represents the SEM; *n* = 30 responding cells from two independent cultures. ∗∗∗*p* < 0.001 and mixed ANOVA. (G) Western blots indicate reduced phosphorylated Mek1/2 (p-Mek1/2) and Erk1/2 (p-Erk1/2) in primary mouse cortical neurons. Neurons were treated with AMPA (1 μM) on DIV12 for 4 h. (H and I) Quantification of western blot data shown in (G). Data of p-Erk1/2 are relative to total Erk1/2 (H), and data of p-Mek1/2 are relative to total Mek1/2 (I). Values are presented as mean ± SD. ∗*p* < 0.05; ∗∗*p* < 0.01; ∗∗∗*p* < 0.001; ∗∗∗∗*p* < 0.0001; Wilcoxon rank-sum test (two-sided).

The pathway sensors used in the barcoded pathway profiling assay were also coupled to firefly luciferase as a reporter gene in addition to the barcode reporters. This allowed us to validate the barcode results in functional assays in living cells. We isolated primary cortical neurons from *Taok2* cKO and control mice and infected the culture with an adeno-associated virus (AAV) mix of individual sensors for EGR1p, FOSBp, SARE, and CRE. We stimulated these neuron cultures with AMPA, BDNF, or BIC. Luciferase readings confirmed the data from the barcoded pathway profiling assay for these sensors (Figures 2A–2D), supporting the notion that TAOK2 knockout impairs neuronal activity and ERK signaling in particular.

For independent validation of the reduced calcium signaling, we used the genetically encoded, green fluorescent protein (GFP)-based GCaMP calcium sensor 6f (GCaMP6f).^29^ Before stimulation, both control and *Taok2* cKO neurons exhibited similar levels of GCaMP6f fluorescence, indicating comparable initial cytosolic calcium concentrations. Upon stimulation with 25 mM KCl, a rapid increase in fluorescence intensity was observed in control and *Taok2* cKO neurons, reflecting a significant cytosolic calcium influx. In *Taok2* cKO neurons, this increase in fluorescence intensity was markedly lower compared to control neurons (Figure 2E). In addition, control neurons consistently showed higher peak fluorescence intensities compared to KO neurons across all time points after the addition of KCl (Figure 2F). To validate the impact of TAOK2 on the ERK branch of MAPK signaling, we next quantified phosphorylation levels of ERK by western blot. Following a 5-min stimulation with AMPA, phosphorylation of Erk1/2 was significantly decreased in primary mouse cortical *Taok2* cKO neuron culture compared to control cultures (Figures 2G and 2H). Similarly, the relative change in phosphorylation of Mek1/2—the upstream kinases of Erk1/2—^30^ was lower in *Taok2* cKO neurons both at baseline and after AMPA stimulation (Figures 2G and 2I). In summary, our results indicate a dysregulation of both ERK/MAPK and calcium pathways in *Taok2* cKO neurons, uncovering a role of TAOK2 in modulating key signaling cascades of synaptic plasticity.

### Taok2 cKO neurons display altered morphology and exhibit reduced synaptic density

**Figure 3:**
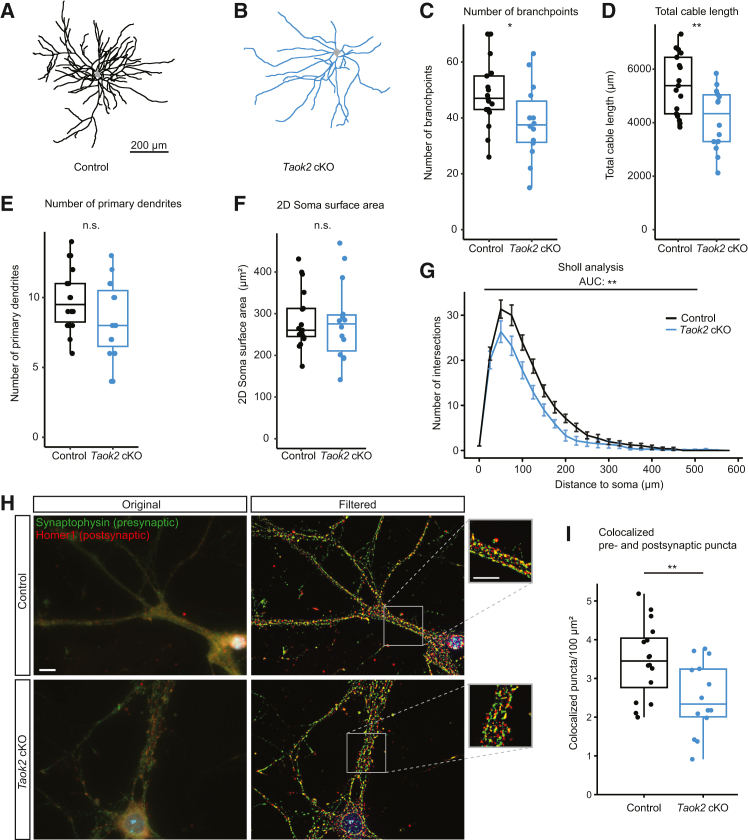
Synapse density and network complexity were reduced in *Taok2* cKO neurons (A and B) Example images of control and *Taok2* cKO neurons. Light gray area: soma of neurons. (C) Quantitative analysis shows that the number of branchpoints was significantly lower in *Taok2* cKO (*n* = 14) compared to control (*n* = 17); *p* = 0.038; Student’s t test (two-sided). (D) The total neuritic cable length was significantly shorter in *Taok2* cKO (*n* = 14) compared to control (*n* = 17); *p* = 0.004; Student’s t test (two-sided). (E) Non-significant decrease of primary dendrites in *Taok2* cKO (*n* = 15) compared to control (*n* = 18); *p* = 0.091; Student’s t test (two-sided); n.s., not significant. (F) 2D soma surface area was not significantly different between control (*n* = 17) and *Taok2* cKO (*n* = 14) neurons; *p* = 0.83; two-tailed Student’s t test with Welch’s correction. (G) Sholl analysis of *Taok2* cKO neurons (*n* = 14) and controls (*n* = 17) shows a reduced dendritic complexity in mutants. Data are presented as mean ± SEM. The area under the curve (AUC) was calculated and compared between groups; *p* = 0.002, with two-tailed Student’s t test. (H) Immunostaining of neurons. Control and *Taok2* cKO neurons stained with presynaptic marker synaptophysin in green (chicken, Synaptic Systems, Cat# 101006, 1:500 dilution; secondary antibody, Alexa-488, 1:500) and postsynaptic marker homer1 in red (rabbit, Synaptic Systems, Cat# 160003, 1:500 dilution for ICC, secondary antibody AB215, Alexa-647, 1:500). Right images and insets: high-pass filtered (ImageJ, FFT band-pass, 0–15 pixels; autoscaled and saturated). Scale bars, 10 μM. (I) Boxplot showing quantitative analysis of colocalized puncta. There is a significant difference in the number of colocalized puncta between *Taok2* cKO and control neurons (*p* = 0.0096, Student’s t test, two-sided). Puncta Analyzer plugin was used in ImageJ to count synaptic puncta. Sample size: *n* = 10 for both *Taok2* cKO and control groups. See also Figure S3.

Next, we assessed the impact of TAOK2 depletion on neuronal morphology to further characterize its role in synaptic structure and function. A fraction of the freshly prepared mouse primary cortical neurons was infected with AAV to express enhanced GFP (EGFP). Labeled neurons were co-plated at a ratio of 1:1,000 with non-infected neurons of the same genotype to minimize dendritic overlap. Neuron morphology of *Taok2* cKO neurons and controls was imaged using a two-photon microscope (Figures 3A and 3B; Figure S3). *Taok2* cKO neurons exhibited fewer branch points (Figure 3C) and reduced the total neuritic cable length (Figure 3D), while no significant differences were detected for primary neurite numbers (Figure 3E) and soma size (Figure 3F). The dendritic complexity in *Taok2* cKO neurons, as measured by Sholl analysis, was distinctly decreased (Figure 3G). These results suggest that *Taok2* knockout specifically affects the dendritic architecture. Synapse density was measured by immunofluorescence, analyzing colocalization of presynaptic (synaptophysin) and postsynaptic (homer1) markers. The number of overlapping puncta stained for synaptophysin and homer1 was significantly reduced in *Taok2* cKO neurons (Figures 3H and 3I). These findings demonstrate a clear effect of TAOK2 on neuronal connectivity, dendritic morphology, and synapse numbers in neuron cultures.

### Single-nucleus transcriptomics identifies changes in excitatory neurons

**Figure 4:**
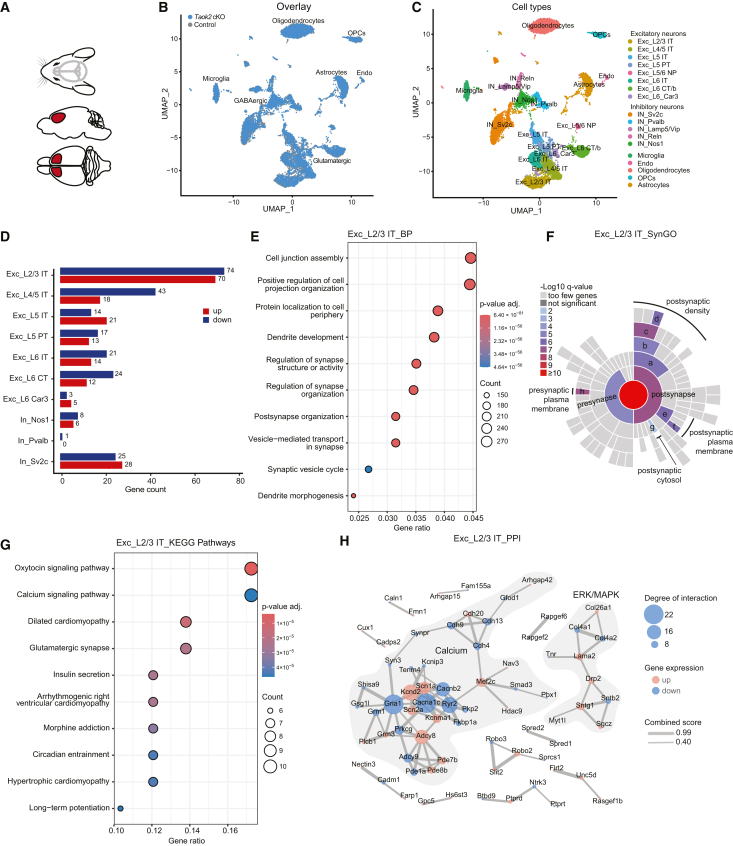
Single-nucleus RNA-seq identified differentially expressed genes enriched in synaptic signaling pathways in neuronal cell types (A) The medial prefrontal cortex (mPFC) was analyzed by snRNA-seq. (B) UMAP plot of *Taok2* cKO and control mice are based on integrated analysis of mPFC snRNA-seq datasets. (C) Eighteen distinct cell types that include 13 clusters of neuronal cell types are visualized by UMAP. Exc, excitatory neurons; In, inhibitory neurons; CT, corticothalamic; endo, endothelial; ET, extratelencephalically projecting; IT, intratelencephalically projecting; micro, microglial cell; NP, near-projecting; OPC, oligodendrocyte precursor cell; PT, pyramidal tract. (D) Significantly different expression gene counts in neurons, adjusted *p* value <0.05 (Wilcoxon rank-sum test) and average log2 fold change >0.25. (E) Dot plots showing the enrichment of gene sets associated with synaptic structures in excitatory neurons of layer 2/3 (Exc_L2/3 IT). Enriched GO terms in the Biological Process (BP) category were identified from the list of significant differentially expressed genes (DEGs). DEGs of Exc_L2/3 IT neurons were analyzed using the *clusterProfiler* package for Gene Ontology (GO) functional enrichment using pathway overrepresentation analysis (ORA). (F) SynGO analysis using GO annotation “Cellular Component” of the significant DEGs of layer 2/3 excitatory neurons. a, postsynaptic specialization; b, postsynaptic density; c, postsynaptic density membrane; d, integral component of postsynaptic density membrane; e, postsynaptic membrane; f, integral component of postsynaptic membrane; g, postsynaptic cytosol; h, integral component of presynaptic membrane. (G) Significantly enriched pathways were identified using the KEGG database. The *x* axis represents the gene ratio (number of significant DEGs in the pathway divided by total number of significant DEGs); the *y* axis lists enriched pathways. Dot size indicates the number of genes involved in each pathway, and color represents the adjusted *p* value (FDR). (H) Protein-protein interaction networks based on the STRING database. The line thickness indicates the strength of data support for each interaction. The minimum required interaction score was set to a medium confidence of 0.4. The degree of a node is defined as the number of edges linking to it. The edge width is based on a combined score integrating data from experimental and predicted interactions. See also Figures S4–S7 and Tables S2, S3, S4, S5, S6, and S7.

To study cell-type-specific effects of *Taok2* depletion in the brain, we conducted snRNA-seq using mouse medial prefrontal cortex (mPFC) tissue (Figure 4A). snRNA-seq of *Taok2* cKO and control samples identified seven major cell types and showed a high overlap for the two genotypes, suggesting that the *Taok2* cKO does not have a general effect on brain development and cell differentiation (Figure 4B). A more detailed cluster analysis revealed 18 distinct cell types, including eight clusters of excitatory neuron subtypes, five clusters of inhibitory neuron subtypes, and five clusters of non-neuronal cell types (Figure 4C; Figures S4A–S4C; Table S2).^31^^,^^32^

By far largest number of differentially expressed genes (DEGs) between the genotypes was seen in excitatory neurons of cortical layer 2/3 (Figure 4D; Table S3). This suggests that these layers are particularly sensitive to *Taok2* inactivation. Pathway overrepresentation analysis (ORA) of significantly downregulated *Taok2* cKO DEGs in layer 2/3 excitatory neurons against the well-annotated Gene Ontology (GO)-based pathway subcollection “Biological Process” (GO-BP) identifies an enrichment in pathways related to the nervous system and synaptic function (Figure 4E). To analyze the synaptic function of significant DEGs in the layer 2/3 neurons in more detail, we used the expert-curated Synaptic Gene Ontologies (SynGO) resource. Functions affected by the *Taok2* knockout were predominantly enriched at the postsynapse (Figure 4F). In addition, a KEGG pathway analysis using significant DEGs in layer 2/3 identifies genes associated with MAPK and calcium signaling as well as glutamatergic and GABAergic synapses (Figure 4G; Table S4). When analyzing the protein-protein interactions underlying the significant *Taok2*-regulated DEGs in layer 2/3 neurons, we identified several proteins that play a role in synaptic signaling and impact ERK and calcium pathways (Figure 4H). Most notably, we observed a robust overall downregulation of several genes that are integral to synaptic plasticity as well as GABA_A_ receptor activity and function. In particular, Ca^2+^/calmodulin-dependent protein kinase II alpha (Camk2a), a key calcium-regulated enzyme that regulates excitatory and inhibitory synaptic plasticity,^33^ was downregulated. Furthermore, adenylate cyclase 8 (Adcy8), which is relevant for synaptic plasticity by modulating long-term memory and long-term potentiation through the CREB transcription factor^34^; myocyte enhancer factor 2C (Mef2c), a calcium-regulated transcription factor^35^; and laminin subunit alpha 2 (Lama2), which encodes the α subunit of extracellular laminin,^36^ are implicated in calcium and ERK signaling and were downregulated in *Taok2* cKO neurons. In addition, we identified other key proteins involved in synaptic signaling as members of the TAOK2-regulated network, including the ionotropic AMPA glutamate receptor subunit 1 (Gria1), the ionotropic NMDA glutamate receptor subunit 2B (Grin2B), and the postsynaptic density scaffolding protein homer1, that all contribute to synaptic calcium signaling. Similarly, layer 4/5 excitatory neurons also show an enrichment in DEGs associated with GO terms related to synapse function, with a particular enrichment in the postsynapse (Figure 4D; Figures S5A and S5B; Table S3). Layer 4/5 DEGs are also implicated in glutamatergic calcium signaling pathways (Figures S5C and S5D). Next, we conducted a gene set enrichment analysis (GSEA) as an alternative methodology for the assessment of pathway enrichment (Figure S6A). GSEA of the whole snRNA-seq dataset detected synaptic functions in both layer 2/3 neurons and layer 4/5 neurons (Figures S6B–S6D; Table S5) in agreement with the ORA analyses for DEGs described above. Interestingly, of the known direct protein-protein interactors of TAOK2 from the BioGRID database,^37^ *CLMN*, *CNKSR2*, *GRIA2*, *GRIN2B*, and *NDRG3* were also DEGs (Table S6), and each of these is implicated in synaptic organization and neurotransmission.

### Taok2 cKO in excitatory neurons leads to indirect expression changes in inhibitory neurons

Inhibitory neurons, which did not have *Taok2* inactivated, showed an enrichment in neuronal and synaptic pathways based on ORA analysis of significant DEGs (Figure S5E; Table S5). Interestingly, synaptic DEGs once again clustered predominantly to the postsynapse based on the SynGO analysis (Figure S5F). Also, significant DEGs from these interneurons are implicated in synaptic signaling based on KEGG pathway analysis (Figure S5G), but showed lower degree of clustering (Figure S5H). Finally, GSEA for DEGs from interneurons also resulted in an enrichment for neuronal and synaptic pathways (Figure S6E; Table S5). The Cre recombinase under the control of the *Emx1* promoter used for creating the *Taok2* cKO mice has been reported to be exclusively active in the excitatory-neuron lineage.^25^ Nevertheless, inhibitory neurons were indirectly affected, with upregulated DEGs that are again enriched for postsynaptic organization and function. We also identified sporadic DEGs in astrocytes, microglia, oligodendrocytes, and oligodendrocyte progenitors (Table S3). However, we could not detect any enrichment of DEGs for GO annotated pathways using ORA or GSEA in any of these non-neuronal cell types. Taken together, we found that the *Taok2* inactivation in excitatory neurons affects mostly postsynaptic function in excitatory as well as inhibitory neurons, suggesting that network changes in activity across different neuronal cell types and cortical layers causes the overall functional phenotypes.

### snRNA-seq is more sensitive than bulk RNA-seq for complex brain tissue

Previously, bulk RNA-seq of mouse cortices with a constitutive homozygous *Taok2* knockout was reported.^38^ However, this approach did not detect any significant GO term gene sets for synaptic organization or activity (Figure S7A). We compared our snRNA-seq-derived DEGs, comprising 137 uniquely upregulated and 163 uniquely downregulated DEGs found in neuronal cell types, with the previous DEGs, which included 203 upregulated and 182 downregulated genes. No overlap was observed among upregulated DEGs, and only two genes overlapped among downregulated DEGs (Figures S7B and S7C; Table S7). The minimal overlap observed between the two datasets demonstrates that cell-type specific effects in a complex tissue might only be identified by snRNA-seq.

### TAOK2-dependent transcriptional dysregulation and its association with mental disorders

To assess association of the TAOK2-dysregulated genes with mental disorders, we queried significant DEGs enriched in both layer 2/3 and layer 4/5 neurons using the DisGeNET database.^39^ Each DEG was searched for in combination with the term “mental disorders” and then ranked according to the gene-disease association score provided by the database. Multiple DEGs from both cortical layers are indeed associated with mental disorders, including autism, schizophrenia, bipolar disorder, Alzheimer’s disease, and neurodevelopmental disorders (Figure S8), confirming that TAOK2 is part of a network that is highly relevant to these neuropsychiatric disorders.

### Conditional Taok2 inactivation in excitatory neurons leads to an anxiety-like phenotype in mice

**Figure 5:**
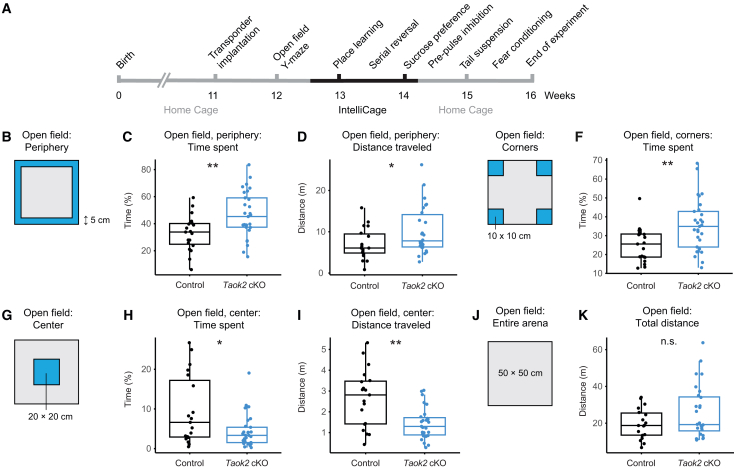
Behavioral profiling of *Taok2* cKO mice identified an anxiety phenotype (A) Timeline of the behavioral tests of the PsyCoP platform. (B) Open field arena: periphery (outermost 5 cm; blue). (C) *Taok2* cKO mice spent more time in the periphery. *p* = 0.0084, two-way ANOVA with Tukey’s post-hoc test. (D) *Taok2* cKO showed a significant increase in distance traveled in the periphery. *p* = 0.038, two-way ANOVA with Tukey’s post-hoc test. (E) Open field arena: corners (10 × 10 cm; blue). (F) *Taok2* cKO mice spent significantly more time in the corners compared to controls. *p* = 0.0028, two-way ANOVA test with Tukey’s post-hoc test. (G) Open field arena: center (20 × 20 cm; blue). (H) *Taok2* cKO mice spent less time in the center. *p* = 0.04, with two-way ANOVA test with Tukey’s post-hoc test. (I) *Taok2* cKO traveled less distance in the center. *p* = 0.0026, with two-way ANOVA test with Tukey’s post-hoc test. (J) Open field arena: entire area (50 × 50 cm). (K) *Taok2* cKO and controls traveled comparable distances in the entire open field arena. *p* = 0.27, Wilcoxon rank-sum test. Cohort size: control, *n* = 19; *Taok2* cKO, *n* = 29. ∗*p* ≤ 0.05; ∗∗*p* ≤ 0.01; n.s., not significant. See also Figures S9–S11.

To explore whether these significant differences observed in neuron culture and brain tissue also translate to the behavioral level, we profiled *Taok2* cKO mice and littermate controls in our semi-automated behavior test and analysis platform termed PsyCoP. This platform integrates multiple behavioral tests and covers the phenotypic domains defined by the RDoC classification,^40^ and is regularly used in our lab for scoring phenotypes relevant to neuropsychiatric conditions in mice (Figure 5A).^41^ The most obvious result from this test battery was an increase in anxiety-related behavior in the open field test. Mice naturally show thigmotactic behavior and prefer to stay close to walls, particularly in well-lit environments.^42^ To evaluate this anxiety-related behavioral domain, we routinely perform the open field test under regular room light. *Taok2* cKO mice spent significantly more time in the periphery next to the wall (outmost concentric 5 cm zone; Figures 5B and 5C) and traveled longer distances in this zone (Figure 5D). When analyzing the distance from the wall in 5 cm increments, *Taok2* cKO mice preferred to keep direct contact to the walls (strip 1) but displayed no genotype differences for strips 2 and 3 (Figure S9). *Taok2* cKO mice also spent more time in the four corners (Figures 5E and 5F). In contrast, *Taok2* cKO spent less spent time in the open field center and also traveled less in this zone (Figures 5G–5I). Overall, there was no group difference for the total distance traveled (Figures 5J and 5K). Both groups also showed no differences for distance traveled in the two inner concentric zones (Figure S9). In addition, fecal pellet number was slightly, albeit not significantly, higher for *Taok2* cKO mice in the open field, further supporting an anxiety-like phenotype (Figure S10A). No additional differences were observed in any other behavioral tests (Figures S10B–S10T). All genotype differences remained significant when analyzing the sexes separately (Figure S11). Taken together, the increased thigmotaxis and increased peripheral locomotion, without affecting overall locomotor activity, suggest a highly specific effect of the *Taok2* knockout on anxiety-related behavior.

## Discussion

TAOK2 is a well-established risk gene for mental and neurodevelopmental disorders and has previously been studied in neuronal cell culture models as well as in constitutive knockout mice.^24^^,^^43^^,^^44^ However, the specific neuronal signaling pathways and cellular and regulatory mechanisms through which TAOK2 influences anxiety-related phenotypes remain incompletely understood. To address this knowledge gap, we have, for the first time, generated a conditional mouse model in which *Taok2* is specifically inactivated in excitatory neurons while remaining intact in inhibitory neurons. Using this experimental model, we established primary cortical neuron cultures and employed a comprehensive cellular signaling profiling assay, which revealed TAOK2-dependent changes in calcium and ERK signaling. In addition, we observed substantial changes in dendritic architecture and synaptic morphology. Using snRNA-seq of prefrontal cortical brain tissue, we further uncovered novel gene networks that are controlled by TAOK2.

### TAOK2 modulates ERK/MAPK and calcium signaling

To elucidate the impact of *Taok2* deletion on intracellular signaling, we used the barcoded pathwayProfiler assay, which enables multiplexed profiling of synthetic pathway sensors in living primary mouse cortical neurons.^26^ This approach identified the ERK branch of the MAPK pathway and calcium signaling as major pathways disrupted in *Taok2* cKO neurons. Particularly, both ERK-specific pathway sensors, such as EGR1p and FOSp, and calcium-specific sensors, such as CRE and SARE, exhibited significantly reduced activity in stimulated *Taok2* cKO neuron cultures compared to wild-type controls. These findings extend earlier reports linking TAOK2 to the JNK and p38 MAPK pathways in neurons^24^ and provide a mechanistic basis for the impaired synaptic connectivity and plasticity.^14^^,^^24^ Our snRNA-seq results of prefrontal cortex tissue isolated from *Taok2* cKO and control mice supported these findings by revealing that synaptic gene networks, particularly those related to calcium, and to a lesser extent, ERK/MAPK signaling, were significantly dysregulated. GO term enrichment analysis indicated that the postsynapse was most strongly affected. Notably, the pathwayProfiler assay and snRNA-seq complement each other and measure different cellular processes: the pathwayProfiler assay captures the rapid, stimulus-induced dynamics of intracellular signal transduction cascades, whereas snRNA-seq reflects longer-term transcriptional adaptations, including synapse reorganization.

Given its methodological simplicity, scalability, and cost-effectiveness, the pathwayProfiler assay represents a compelling alternative or complement to conventional RNA-seq techniques, especially for studies involving numerous experimental conditions, such as dose-response stimulation paradigms. Unlike RNA-seq, the pathwayProfiler assay requires a lower sequencing depth and enables high-dimensional multiplexing. Its synthetic sensors are based on the promoter sequences of immediate-early response genes, such as EGR1, FOS,^45^ FOSB, and NR4A1.^46^ All these sensors robustly responded to AMPA, BDNF, and BIC stimulation in primary neuron cultures and reliably detected the genetic *Taok2* deficit. In addition to sensors designed for capturing neuronal activity, the pathwayProfiler sensor library also contains sensors for proliferation, metabolism, immune signaling, and cellular stress, underscoring its broad applicability across diverse cell types and disease models. In this study, we have analyzed four 96-well plates with 48 barcodes per well in a single sequencing run but estimate that up to 100 plates and twice as many barcodes could be multiplexed within a single experiment.

### TAOK2 regulates calcium flux

The pathwayProfiler assay revealed an attenuated calcium response in *Taok2* mutant neurons as measured by reduced CRE and SARE sensors activity. We validated this observation using the genetically encoded calcium sensor GCaMP6f, which confirmed a decreased calcium influx following KCl stimulation in *Taok2* cKO neuron cultures. Calcium and ERK/MAPK signaling are closely linked cellular processes. Elevated intracellular calcium activates the ERK pathway, often through effectors like calmodulin-dependent kinases or protein kinase C. In neurons, calmodulin is activated by calcium influx, e.g., via L-type voltage-dependent calcium channels, synaptic NMDA receptors, or release from intracellular stores. Activated calmodulin, in turn, stimulates Ras GTPases, triggering the ERK cascade and downstream transcriptional responses.^47^ Consistent with this mechanism, previous work identified TAOK2 as a regulator of cytosolic calcium influx,^19^ a finding that we now extend to excitatory neuron-specific knockout models.

### Single-nucleus RNA sequencing identifies TAOK2-regulated gene networks

Single-cell RNA-seq and snRNA-seq enable high-resolution detection of differential gene expression while preserving cellular context,^48^ making these methods particularly valuable for studying complex tissues such as the brain. In this study, *Taok2*-regulated DEGs were highly enriched for postsynaptic gene sets and formed protein-protein interaction networks centered on for calcium and ERK/MAPK signaling. Notably, prior bulk RNA-seq analyses of cortices from *Taok2* constitutive full knockout mice did not detect these signatures,^38^ highlighting the added resolution afforded by snRNA-seq. Disease association analyses revealed significant enrichment of *Taok2*-regulated DEGs in gene sets linked to psychiatric disorders, particularly autism and schizophrenia. This is consistent with the established roles of excitatory neuron dysfunction in the etiology of these disorders,^49^^,^^50^^,^^51^^,^^52^ wherein altered gene expression perturbs pathways critical for synaptic function and development.^53^^,^^54^ Interestingly, despite the excitatory neuron-specific knockout, we observed transcriptional changes in inhibitory neurons, potentially reflecting altered network activity. This is plausible, given that inhibitory neurons provide critical feedback and feedforward inhibition to excitatory neurons,^55^ implying that reduced excitatory output could indirectly modulate inhibitory neuronal activity.

### TAOK2 controls dendritic complexity and synapse density

The signaling and transcriptomic changes associated with *Taok2* deletion were accompanied by pronounced morphological deficits. *Taok2* cKO neurons exhibited reduced dendritic complexity, evidenced by fewer dendritic branch points, shorter neuritic cable length, and a decreased dendritic intersections. Immunocytochemical analyses revealed a concomitant reduction in synapse density. These observations are consistent with prior RNA interference studies that also showed reduced dendritic branching.^14^ Likewise, neurons from constitutive *Taok2* knockout mice exhibited reduced dendritic complexity and synaptic connectivity.^24^ Conversely, overexpression of Taok2, either by transient transfection^12^ or via duplication of the 16p11.2 locus,^56^ resulted in increased dendritic complexity. This effect is mediated in part by ERK signaling, as pharmacological inhibition of ERK in neurons with the 16p11.2 duplication reduced dendritic complexity across the arbor.^57^ While the 16p11.2 locus also contains other ERK pathway components, such as MAPK3 (encoding ERK1), our findings clarify that TAOK2 itself is a key regulator of this pathway in excitatory neurons. The primary neuron cultures examined in this study, derived from embryonic day 15.5 (E15.5) embryos, are predominantly composed of layer 2/3 pyramidal neurons.^24^^,^^58^ The observed morphological phenotypes are consistent with previous *in situ* analyses of layer 2/3 neurons in constitutive homozygous *Taok2* knockout mice^58^ and with developmental studies demonstrating that TAOK2 specifically influences upper cortical layer formation.^18^

### Taok2 inactivation increases anxiety-like thigmotactic behavior

Behaviorally, *Taok2* cKO mice showed significantly increased thigmotactic behavior, characterized by a preference for remaining close to the arena walls under well-lit conditions in the open field test, with increased peripheral locomotion, but without changes in total locomotor activity. This anxiety-like behavior was highly specific, as none of the other tests in our phenotyping battery PsyCoP revealed an anxiety-relevant phenotype. Notably, we detected this anxiety-like phenotype during the first 10 min in the open field test, a finding that was previously not detected at time points of up to 30 min using constitutive knockouts.^24^ We speculate that the elevated anxiety response we observed during the first 10 min may mask the reductions in border time the other authors reported for later time points. The cell-type-dependent inactivation of *Taok2* in excitatory neurons thus appears sufficient to elicit a robust anxiety-like phenotype, as observed by the strong thigmotactic behavior. This aligns with our observation of impaired ERK/MAPK signaling, given that ERK1/2 pathway modulates anxiety-like behaviors in mice.^59^^,^^60^ Notably, the anxiolytic neuropeptide oxytocin can enhance phosphorylation of RAF1, MEK1/2, and ERK1/2, thereby exerting anxiolytic effects.^61^

While previous research has linked TAOK2 to neuronal stress pathways including JNK and p38 MAPK,^24^ our data demonstrate that TAOK2 also regulates the ERK branch, which is directly linked to synaptic function.^22^ In summary, all our findings support the notion that TAOK2 modulates calcium and ERK/MAPK signaling, which in turn influence neuronal activity, dendritic and synaptic architecture, and anxiety-related behaviors. Given that TAOK2 is a validated risk gene for autism,^5^ schizophrenia,^3^ and Alzheimer disease,^8^^,^^9^ pharmacological targeting of TAOK2 or its downstream pathways may offer a promising therapeutic avenue for these neurodevelopmental and neuropsychiatric disorders.

### Limitations of the study

Taok2 was selectively inactivated in glutamatergic neurons. The pathwayProfiler assay, as well as standard single-luciferase reporter assays and western blot assays, only measures bulk network activity from neuron cultures and does not distinguish between excitatory and inhibitory neurons. Nevertheless, interneuron functionality was confirmed using bicuculline, which strongly increased MAPK and calcium sensor signals. As ∼80%–90% of neurons in our cultures are excitatory, most signaling activities reflect this population. Another limitation of this study is that anxiety-like behavior was assessed only with the open field test. *Taok2* cKO mice exhibited a strong and robust thigmotaxis phenotype in this test, a measure of anxiety. However, while this assay provides an initial measure of exploratory activity and anxiety-like traits, it does not fully capture the complexity of anxiety-related behaviors. Additional paradigms such as the elevated plus maze, light-dark box, or novelty-suppressed feeding should be used in future work to validate and extend the current findings. So far, TAOK2 has only been inactivated in all cells using a complete knockout,^24^^,^^44^ and we now provide data for its selective inactivation in excitatory cortical neurons. To fully understand TAOK2’s role in neuronal network function, future studies could selectively inactivate it in other neuronal cell types, such as inhibitory neurons or glial cells, to broaden the generalizability of our findings.

## Resource availability

### Lead contact

Further information and requests for resources and reagents should be directed to and will be fulfilled by the lead contact, Michael C. Wehr (michael.wehr@med.uni-muenchen.de).

### Materials availability

Plasmids used in standard assays are available from Addgene. Addgene IDs are listed in the key resources table.

There are restrictions to the availability to the viral plasmids used in the pathwayProfiler assay due to intellectual property considerations.

### Data and code availability

Data of the pathwayProfiler assay, orthogonal validation assays, single-nucleus RNA sequencing, and behavioral profiling have been deposited at Mendeley Data and is publicly available as of the date publication. DOIs are listed in the key resources table.

Original code has been deposited at Mendeley Data and is publicly available as of the date of publication. DOIs are listed in the key resources table.

## Acknowledgments

We thank Beate Kauschat and Monika Rübekeil for excellent technical support. The research was supported through the China Scholarship Council-LMU Munich program, grant number 202108620046 awarded to W.M. Open access publication funding was provided by 10.13039/501100005722LMU Munich.

## Author contributions

Conceptualization, W.M. and M.C.W.; data curation, W.M., B.B., and M.C.W.; formal analysis, W.M., I.W., and N.K.; methodology, W.M., I.W., X.M., K.D., N.J., and M.C.W.; investigation, W.M., I.W., X.M., M.S., P.V., and M.C.W.; visualization, W.M., I.W., N.K., and M.C.W.; resources, M.J.R., V.S., and M.C.W.; supervision: V.S. and M.C.W. Writing – original draft: W.M. and M.C.W. Writing – review & editing: all authors.

## Declaration of interests

B.B., M.J.R., and M.C.W. are employees and shareholders of Systasy Bioscience GmbH, Planegg, Germany.

## STAR★Methods

### Key resources table

**Table:** 

REAGENT or RESOURCE	SOURCE	IDENTIFIER
**Antibodies**		

Goat polyclonal anti-TAOK2	Santa Cruz Biotechnology	Cat# sc-47447; RRID:AB_2240280
Phospho-p44/42 MAPK (Erk1/2) (Thr202/Tyr204) (D13.14.4E) XP	Cell Signaling Technology	Cat# 4370; RRID:AB_2315112
p44/42 MAPK (Erk1/2) (137F5)	Cell Signaling Technology	Cat# 4695; RRID:AB_390779
Phospho-MEK1/2 (Ser217/221) (41G9)	Cell Signaling Technology	Cat# 9154; RRID:AB_2138017
MEK1/2 (D1A5)	Cell Signaling Technology	Cat# 8727; RRID:AB_10829473
Synaptophysin1	Synaptic systems	Cat# 101 009; RRID:AB_3662005
Homer 1	Synaptic systems	Cat# 160003; RRID:AB_887730
Mouse monoclonal anti-alpha-tubulin	Sigma-Aldrich	Cat# T5168; RRID:AB_477579
Peroxidase-AffiniPure goat anti-mouse IgG (H + L)	Jackson Immuno Research Labs	Cat# 115-035-003; RRID:AB_10015289
Peroxidase-AffiniPure F(ab')2 fragment goat anti-rabbit IgG (H+L)	Jackson Immuno Research Labs	Cat# 111-036-003; RRID:AB_2337942
Goat polyclonal anti-chicken IgY (H+L) secondary antibody, Alexa Fluor 488	Thermo Scientific	Cat# A-11039; RRID:AB_2534096
Goat polyclonal anti-rabbit IgG (H+L) cross-adsorbed secondary antibody, Alexa Fluor 647	Thermo Scientific	Cat# A-21244; RRID:AB_2535812

**Bacterial and virus strains**		

*Escherichia coli* One Shot Mach1 competent cells	Thermo Fisher Scientific	Cat# C862003
*Escherichia coli* One Shot Stbl3 chemically competent cells	Thermo Fisher Scientific	Cat# C737303

**Chemicals, peptides, and recombinant proteins**		

QIAzol lysis buffer	Qiagen	Cat# 79306
Direct-zol RNA MiniPrep kit	Zymo	Cat# R2052
High-capacity cDNA reverse transcription kit	Thermo Fisher Scientific	Cat# 4368813
Passive lysis buffer	Promega	Cat# E1941
Firefly luciferase substrate: d-luciferin, free acid	PJK GmbH, Germany	Cat# 102112
Firefly luciferase substrate: coenzyme A	PJK GmbH, Germany	Cat# 102212
Firefly luciferase substrate: DTT	PJK GmbH, Germany	Cat# 102252
DMEM, 4.5 g/L glucose medium	Thermo Fisher Scientific	Cat# 11880028
Fetal Bovine Serum (FBS), qualified, heat inactivated, Brazil	Thermo Fisher Scientific	Cat# 10500064
GlutaMAX Supplement	Thermo Fisher Scientific	Cat# 35050038
EverBrite hardset mounting medium with DAPI	Biotium	Cat# 23004
Benzonase	Sigma	Cat# E1014
Neurobasal medium	Thermo Fisher Scientific	Cat# 21103049
B-27 Supplement (50x)	Thermo Fisher Scientific	Cat# 17504044
Papain	Cell Systems	Cat# LS 003126
l-Cysteine	Sigma-Aldrich	Cat# C7880
DNase I	Cell Systems	Cat# DN25
HBSS (no calcium, no magnesium, no phenol red)	ThermoFisher Scientific	Cat# 14175-053
OptiPrep	StemCell Technology	Cat # 07820

**Critical commercial assays**		

Protein assay dye reagent concentrate for Bradford method	Bio-Rad	Cat# 500-0006
NovaSeq 6000 SP reagent kit v1.5 (100 cycles)	Illumina	Cat# 20028401
Ion PI chip kit v3	Ion Torrent/Thermo Fisher Scientific	Cat# A26771 (discontinued)
Qubit dsDNA assay kit	Thermo Fisher Scientific	Cat# Q32851
GoTaq G2 Flexi DNA polymerase kit	Promega	Cat# M7805

**Deposited data**		

Data of pathwayProfiler assay	This paper	Mendeley Data: https://doi.org/10.17632/k2n7jwx9rn.1
Data of luciferase assays	This paper	Mendeley Data: https://doi.org/10.17632/k2n7jwx9rn.1
Data of snRNA-seq	This paper	Mendeley Data: https://doi.org/10.17632/k2n7jwx9rn.1
Heatmap R script	This paper	Mendeley Data: https://doi.org/10.17632/k2n7jwx9rn.1
snRNA-seq R script	This paper	Mendeley Data: https://doi.org/10.17632/k2n7jwx9rn.1

**Experimental models: Cell lines**		

293T cells	ATCC	Cat# CRL-3216; RRID:CVCL_0063

**Experimental models: Organisms/strains**		

Taok2^tm1a(EUCOMM)Hmgu^ ES cell clone	EUCOMM	Cat# HEPD0565_3_H03
*Taok2 (fl/fl)* mice (B6N-A^*tm1Brd*^*Taok2*^*tm1c(EUCOMM)Hmgu*^/Mjro)	This paper	MGI:4434943, EMMA:11308
*Emx1-Cre* mice (B6N-Emx1^tm1(cre)Krj^/J)	Gorski et al.^25^	MGI:2684610, JAX:005628
Primary murine cortical neurons from *Taok2 (fl/fl) x Emx1-Cre* mice	This paper	N/A

**Oligonucleotides**		

Oligos for genotyping, see Table S5	This paper	N/A

**Recombinant DNA**		

Plasmid pAAV_hSyn1P_EGFP	This paper, Addgene	Cat# 232171
Plasmid pFdelta6	Addgene	Cat# 232172
Plasmid pAAV2_Rep52-VP1	Addgene	Cat# 232173
Plasmid pAAV1_Rep52-VP1	Addgene	Cat# 232174
Plasmid pAAV-hSyn1-mRuby2-GSG-P2A-GCaMP6f-WPRE-pA	Addgene	Cat# 50943
Plasmids used in pathwayProfiler assays, see Table S1. All plasmids are pAAV-based.	Herholt et al.^26^	N/A

**Software and algorithms**		

RStudio, version 2024.09.1	RStudio	https://rstudio.com/
R Bioconductor, version 4.2.2	Bioconductor	https://www.bioconductor.org/
Fiji (ImageJ), version 1.53	Fiji team	https://fiji.sc/
Puncta Analyzer plugin for ImageJ	Risher et al.^62^	https://pmc.ncbi.nlm.nih.gov/articles/PMC4286724/#SD1-data
Adobe Illustrator CS6	Adobe Inc.	https://www.adobe.com/products/illustrator.html
clusterProfilerWrapper	GitHub	https://github.com/MNB-Lab/clusterProfilerWrapper
MikroWin 2000 Version 4.41	Mikrotek Laborsysteme (1992 – 2007)	https://mikrowin-2000.software.informer.com/download/
SynGO	Synaptic Gene Ontologies and annotation consortium	https://www.syngoportal.org/
BioGRID	Database for physical protein-protein interactions	https://thebiogrid.org/
DisGeNET	Database for human gene-disease associations	https://www.disgenet.com/
LabVIEW 2017	National Instruments, USA	https://www.ni.com/en/shop/labview.html
MATLAB Version R2021b	The MathWorks Inc., Natick, USA	https://www.mathworks.com/products/new_products/release2021b.html

**Other**		

Mini-PROTEAN Tetra vertical electrophoresis cell	Bio-Rad	Cat# 1658004
ChemoStar imager	Intas Pharmaceuticals	N/A
4–20% Mini-PROTEAN TGX precast protein gels	Bio-Rad	Cat# 4561093
Mithras LB 940 multimode plate reader	Berthold Technologies, Germany	Cat# LB 940
NovaSeq 6000 sequencing system	Illumina	Cat# 20012850
Ion Proton system	Ion Torrent/Thermo Fisher Scientific	Cat# A26771 (discontinued)
IntelliCage	TSE Systems	https://www.tse-systems.com/
Open field arena	Volkmann et al.^41^	Custom-made
Y-maze	Volkmann et al.^41^	Custom-made
Tail suspension rack	Volkmann et al.^41^	Custom-made
ANY-maze (for open field, Y-maze, fear conditioning, tail suspension)	Stoelting	http://www.anymaze.com/
Fear conditioning system	Ugo Basile	https://ugobasile.com/products/categories/behaviour-conditioning-reward/fear-conditioning-system
SR-Lab startle response system (for pre-pulse inhibition)	San Diego Instruments	https://sandiegoinstruments.com/product/sr-lab-startle-response/
Zeiss Apotome 3 fluorescence motorized microscope	ZEISS	https://www.zeiss.com/microscopy/us/products/light-microscopes/widefield-microscopes/apotome-3.html
3.5 cm cell culture dish for imaging cells	Eppendorf, Germany	Cat# 0030 700.112
2-Photon microscopic system: Olympus BX51WI fixed stage upright microscope	Olympus, Japan	Cat# BX51WI
LUMPlan x60, 0.9 NA water immersion objective	Olympus, Japan	n. a.
CCD camera for Olympus microscope	Optronis, Germany	Cat# VX45
Mai Tai DeepSee Ti, sapphire laser	Spectra-Physics/Newport, USA	Cat# Mai Tai 10
Electro-optical modulator	Conoptics, USA	Cat# 350-80
Amplifier for electro-optical modulator	Conoptics, USA	Cat# 302RM
2x mechanical shutter	Uniblitz Vincent Associates, USA,	Cat# VMM-D1
2x shutter driver	Uniblitz Vincent Associates, USA	Cat# VCM-D1
2x low-noise current preamplifier	Stanford Research Systems, USA	Cat# SR570
2x photomultiplier tube with D-type socket assembly	Hamamatsu, Japan	Cat# E850-13
SM5 9 control	Luigs & Neumann GmbH, Germany	Cat# Control SM5-9
SM5 remote control system	Luigs & Neumann GmbH, Germany	Cat# SM5-Remote control system
SM5 cube control	Luigs & Neumann GmbH, Germany	Cat# SM5-Cube

### Experimental model and study participant details

#### Mice

Gene-trapped embryonic stem (ES) cells harboring a floxed allele of *Taok2* and a lacZ-NeoR selection cassette (Taok2^tm1a(EUCOMM)Hmgu^; ES cell clone number, HEPD0565_3_H03) were purchased from EUCOMM and injected into a pseudo-pregnant C57BL/6N agouti mouse to yield founder animals. Exons 2 to 11 encode the kinase domain of both transcript isoforms (variant 1, NM_001163774; variant 2, NM_001163775) and exons 5 to 8 were flanked by loxP sites. Cre recombinase mediated excision truncates the kinase domain and leads to a frameshift, resulting in a premature stop in exon 9 (Figure S1A). The lacZ-NeoR cassette was removed by crossing with Flp1 recombinase expressing transgenic mice (B6N.129S4-Gt (ROSA)26Sor^tm1(FLP1)Dym^/J, Jackson Laboratory). Correct integration of the floxed *Taok2* allele was confirmed by long-range PCRs that covered the 5’ and 3’ integration sites of the EUCOMM construct (Figure S1B). *Taok2* was conditionally inactivated in excitatory neurons during brain developing by crossing with *Emx1-Cre* mice.^25^ *Taok2 (fl/fl*, +*)* and *Taok2 (fl/fl) x Emx1-Cre (tg/0)* (*Taok2* cKO) lines were established that, when mated, created homozygous conditional *Taok2* cKO animals and littermate controls (*Taok2 (fl/fl*, +*)*). All mice were group housed under SPF conditions in IVCs for breeding and in open cages during behavior tests under a non-shifted 12:12 light/dark cycle. Behavior tests were performed during the light phase. Water and food were provided *ad libitum*. Animals were kept according to all current rules and regulations and all animal experiments were approved by the State of Upper Bavaria, Germany under the license ROB-55.2-2532.Vet_02-22-110.

#### Primary neuron cultures

E15.5 embryos (*Taok2* cKO and *Taok2 (fl/fl)* controls), were used to prepare primary mouse cortical neuron cultures. Separate cultures were prepared for each embryo, followed by genotyping. Mouse cortices were dissected in cold HBSS supplemented with 5 mM HEPES and treated with activated papain (1 ml DMEM (4.5 g/l glucose) (Thermo Fisher Scientific), 40 μl papain solution (Cell Systems), 40 μl DNaseI (Sigma-Aldrich), and 10 μl l-cysteine (Sigma-Aldrich) at room temperature for at least 10 min until transparent. Papain treatment was terminated with DMEM/FBS medium (DMEM 4.5 g/l glucose, 10 % FBS (Thermo Fisher Scientific)). After washing with DMEM/FBS, cortices were transferred into 1 ml neuronal plating medium (Neurobasal medium (Thermo Fisher Scientific) supplemented with 2 % B27, 5 % FBS, and 1 % GlutaMAX (Thermo Fisher Scientific)) and triturated using a 1000 μl pipet tip. The suspension was filtered through a 40 μm cell strainer (BD Falcon). Cells were seeded at a density of ∼60,000 cells/cm^2^. Cell culture plates were coated with 0.1 mg/ml poly-d-lysine (PDL) diluted in autoclaved ultrapure water (ddH_2_O) overnight, followed by three ddH_2_O washes. On day 1 *in vitro* (DIV1), the plating medium was replaced with neuronal culture medium (Neurobasal medium supplemented with 2 % B27 and 1 % GlutaMAX). Starting on DIV6, half of the medium volume was replaced with fresh neuronal culture medium every 3 – 4 days. The cells were typically lysed on DIV12. Cultures were incubated at 37°C and 5 % CO_2_.

#### Generation of AAVs

15×10^6^ HEK 293T cells (ATCC) were transfected with an equimolar amount of AAV transfer plasmids (ca 5 – 6 μg, depending on the insert size), 7.5 μg of the replication/capsid plasmid (equimolar mixture of serotypes 1 (pAAV1_Rep52-VP1, Addgene Cat# 232174) and 2 (pAAV2_Rep52-VP1, Addgene Cat# 232173)), 10 μg of the pFdelta6 AAV helper plasmid (Addgene Cat# 232172), and 1 μg polyethyleneimine (Polyscience) per 1 μg DNA transfected. After 3 days, cells were scraped, transfered into NaCl/Tris buffer (150 mM NaCl, 50 mM Tris-HCl, pH 8.5), subjected to three freeze/thaw cycles (−80°C for 20 min, followed by 37°C in a water bath), and treated with benzonase (50 U/mL, 30 min, 37°C), followed by centrifugation (10 min at 3000 × g. The virus-containing supernatant was filtered through a 0.45 μM syringe filter, followed by enrichment using Amicon filter units (Millipore) in PBS. Virus preparations were stored in 20 μl aliquots at −80°C and quantified by qPCR using WPRE primers, typically reaching titers of 10^9^ to 10^10^ genomic copies (GC)/μl.

#### Plasmids

The pathwayProfiler assay contains 22 pathway reporters, which are based on synthetic and clustered transcription factor binding sites or human promoter sequences linked to unique barcode reporters. The pathwayProfiler plasmid library comprises 48 pAAV-based plasmids, including 22 different pathway reporters, each represented on two barcoded plasmids, and one control reporter (AAV minimal major late promoter (MLPmin) for calibration of well-to-well differences of raw NGS reads and for normalization of cell numbers, on four separate barcoded plasmids (Table S1). The cloning of the pathwayProfiler plasmids was described previously.^26^

### Method details

#### Genotyping of mice and E15.5 embryos

For genotyping, DNA was isolated from tail tip biopsies using the HotSHOT protocol.^63^ For PCRs, GoTaq G2 Flexi kit (Promega) was used using the standard protocol with 0.1 μM primers, 3 mM (*Taok2*) or 1.5 mM (*Emx1-Cre*) MgCl_2_, 0.025 U/μl GoTaq, and 1 μl genomic DNA in 15 μL reaction volume. Cycler programs: *Taok2*: preheated to 95°C, 1 min 95°C, 37 cycles of 10 s 95°C, 20 s 65°C, and 30 s 72°C, followed by 2 min 72°C and 4°C. Bands: wt 444 bp, fl 634 bp, recombined (rec) 319 bp (Figure S1C). *Emx1-Cre*: preheated to 95°C, 1 min 95°C, 37 cycles of 10 s 95°C, 20 s 55°C, and 30 s 72°C, followed by 2 min 72°C and 4°C. Bands: wt 378 bp, tg 321 bp (Figure S1D). For primer sequences see Table S8.

#### Vector construction

The plasmid pAAV_hSyn1P_EGFP (Addgene Cat# 232171) was cloned by inserting the EGFP open reading frame into a pAAV vector pAAV_hSyn1P under the control of the human Synapsin-1 promoter using BamHI and HindIII.

#### Barcoded pathwayProfiler assay

##### Cell culture, pharmacological treatments, and extraction of mRNA barcode reporters

Cortical primary neurons were seeded into 96-well plates coated with 0.1 mg/ml PDL at a density of 20,000 cells/well and cultured in neuronal culture medium. On DIV2, neurons were infected with the AAV-based pathway sensor library (5,000 GC/cell) after changing half of the medium. Detailed information on the pathway sensors, including response elements and promoters, transcription factor binding, barcode sequences, and corresponding pathways can be found in Table S1. On DIV7, medium was changed, and on DIV 12, neurons were stimulated for 4 h and 24 h with 0.1, 1, 10, or 100 μM of AMPA, BIC, or forskolin, or 0.1, 1, 10, or 100 ng/ml of BDNF. After stimulation, neurons were lysed with 100 μl Tag&Pool lysis/binding buffer (100 mM Tris/HCl, 500 mM LiCl, 10 mM EDTA, 1 % lithium dodecyl sulfate (LiDS), and 5 mM DTT) and stored at −80°C. RNA was then purified with the Dynabeads mRNA Direct Kit (Thermo Fisher Scientific) and cDNA was synthesized using the High-Capacity cDNA Reverse Transcription Kit (Applied Biosystems).

##### Barcode amplification and sequencing

Barcode amplification and sequencing was conducted as previously reported.^64^ In brief, in the first PCR, barcodes were amplified using HotStarTaq Plus DNA polymerase (Qiagen). 1 μl of cDNA was used in a 20 μl PCR reaction (30 cycles, annealing temperature of 59°C) and the products were analyzed by agarose gel electrophoresis. In a second PCR, barcodes were fused with adapter sequences for Ion Torrent sequencing and the reactions were verified by agarose gel electrophoresis. Barcode libraries were sequenced with the Ion PI Sequencing 200 v3 kit (Thermo Fisher Scientific) on an Ion Torrent Proton sequencer (Thermo Fisher Scientific).

##### Analysis of *pathwayProfiler* data

First, based on sample barcodes, sequencing data were split into the original samples, encoding specific treatment conditions and the reads corresponding to assay barcodes. The reads were then trimmed to extract the barcodes and subsequently mapped using a custom script to identify the sensor to which each read was assigned. Each experimental condition consisted of four biological replicates, each with 2 barcodes. Reads mapping to sensor IL6p were removed from further analysis due to baseline expression below thresholds. Differential expression analysis was performed using the *pathPROFILER* package, which wraps the *DESeq2* R package with custom scripts for normalization. Standard procedures for dispersion estimation and statistical testing were applied. Normalization was performed using a scaling factor derived from the MLPmin controls, which served as a non-stimulated reference response. Heatmaps were generated using the *ggplot2* R package based on log2 fold changes (log2FC).

#### Luciferase assays

All assays were run in 96-well plates coated with 0.1 mg/ml PDL and seeded with primary cortical neurons at 20,000 cells/well. On DIV6, neurons were infected with single AAV sensors for EGR1p, FOSBp, SARE, and CRE (prepared from the transfer vectors pAAV_EGR1p, pAAV_FOSBp, pAAV_SARE_MLPmin, pAAV_CRE_MLPmin) at a multiplicity of infection (MOI) of 1000. Each of these sensors drive both a barcode reporter and a luciferase reporter gene. On DIV12, neurons were stimulated for 4 h with AMPA (1 μM), BDNF (10 ng/ml), or BIC (10 μg) at six replicates per condition, lysed in passive lysis buffer (Promega), and analyzed in a luciferase assay using firefly luciferase substrate (PJK GmbH) using the Mithras LB 940 multimode plate reader (Berthold).

#### Western blotting

Primary cortical neurons were plated at a density of 0.6×10^6^ cells/well in 6-well plates. On DIV12, the neurons were stimulated for 5 min with 1mM AMPA and lysed in 1 % Triton-X lysis buffer (50 mM Tris, 150 mM NaCl, 1 % Triton X-100, 1 mM EGTA, *p*H 7.5) supplemented with phosphatase inhibitors (10 mM NaF, 1 mM Na_2_VO_4_, 1 mM ZnCl_2_, and 4.5 mM Na_4_P_2_O_7_) or phosphatase (PhosSTOP, Roche) and protease inhibitor cocktail (cOmpleteMini, Roche) tablets. For Western blots, protein gels (Bio-Rad) were run in a Mini-PROTEAN Tetra Vertical Electrophoresis Cell (Bio-Rad) and gels were blotted using aMini Trans-Blot Cell (Bio-Rad). Proteins were detected by chemiluminescence (ECL Plus substrate, Thermo Fisher Scientific). Images were analyzed in ImageJ. Significances were calculated using the Wilcoxon rank-sum test. For antibody dilutions see Table S9.

#### Measurement of intracellular calcium levels using GCaMP6f

Primary cortical neurons were seeded at a density of 0.5×10^6^ cells/well in 6-well plates coated with 0.1 mg/ml PDL. On DIV1, the medium was replaced with fresh culture medium to remove any non-adherent cells. On DIV6, half the medium was changed and the neurons were infected with AAVs for expression of mRuby2-P2A-GCaMP6f under the control of the human synapsin1 promoter (hSyn1p) (Addgene #50943) at an MOI of 1000. On DIV12, neurons were stimulated with 25 mM KCl under a fluorescence microscope (Axioplan, Zeiss). Videos were recorded for two minutes and analyzed in the ZEN 2 pro software (ZEISS) using a region of interest analyzer (ROI Manager). 15 Responding somata from each of the two independent cultures were analyzed. Five areas from the four corners and the middle of the field of view were used for background normalization. Upon stimulus application, the change in fluorescence intensity of the cells was calculated by subtracting the baseline fluorescence (*ΔF* = *F*_cell_−*F*_baseline_). Genotype, time, and their interaction (time × genotype) effects were analyzed using a linear mixed-effects model as fixed effects with the *lmerTest* package in R. The fitted model was analysed using type III ANOVA with Satterthwaite's method to test the significance of fixed effects.

#### Morphological analysis

To ensure sparse labeling and avoid overlap of GFP-positive neurons, 500 GFP neurons (infected in suspension with pAAV_SynP_EGFP at an MOI of 1000 for 4 h at 37°C) were mixed with 0.5×10^6^ non-infected neurons (1:1000) of the same genotype and co-plated onto 3.5 cm dishes pre-coated with 0.1 mg/ml PDL. On DIV7, one-quarter of the medium was replaced. On DIV14/18 (*Taok2* cKO) or DIV15/19 (*Taok2 (fl/fl)* controls), the morphology of living neurons was analyzed using a 2-photon microscope (see key resources table) with a 60x water-immersion objective at 1024 × 1024 pixels (0.26 μm/pixel) at 488 nm excitation wavelength. Z-stacks at 1 μm increments for several regions of interest per neuron were taken. Neurons were excluded from analysis for blebbing and weak fluorescence or when unambiguous segmentation was not possible. Stacks were stitched using the Stitching Plug-in in ImageJ and morphological parameters were analyzed in ImageJ and the TREES toolbox^65^ in MATLAB. Neurite arbors were traced manually using the Simple Neurite Tracer Plug-in. Arbor complexity was measured in TREES toolbox by Sholl analysis (intersections per concentric circles at 25 μm increments), the sum of all bifurcations, the number of first order dendrites that leave the soma, and the total length of all neurites (total cable length). 2D soma surface area was calculated in ImageJ on maximum intensity projections of the stitched z-stacks using the freehand selection and the measure tool. Sholl data were analyzed using a mixed-effects ANOVA with genotype, distance, and their interaction (genotype × distance) as fixed effects, and replicate as a random effect.

#### Synapse staining and synaptic puncta quantification using immunocytochemistry

Synaptophysin (anti-chicken, Synaptic Systems #101006, 1:500) was used as a presynaptic marker and homer1 (anti-rabbit, Synaptic Systems, #160003, 1:500) as a postsynaptic marker to identify synaptic puncta; colocalization of both markers identifies glutamatergic synapses.^66^^,^^67^ Primary cortical neurons were plated at a density of 5,000 cells/well in 24-well plates containing coverslips that were coated with 0.1 mg/ml PDL. On DIV12, neurons were washed twice with PBS, fixed in cold methanol for 5 min at room temperature, followed by three washes with PBS. Cells were permeabilized with 0.1 % Triton X-100 in Tris-buffered saline (TBS) for 5 min at room temperature, and then blocked in 300 μl blocking solution (0.1 % Triton X-100, 3 % BSA in TBS) for 1 h at RT. Cells were washed three times with TBS and incubated with the primary antibody in 150 μl blocking solution overnight at 4°C. On the next day, cells were washed five times with TBS and incubated with the secondary antibody coupled to Alexa Fluor dyes 488 or 647 (Table S9) in 150 μl blocking solution for 60 min at RT in the dark. Cells were washed three times with TBS and the cover slips mounted on slides (EverBrite hardset mounting medium with DAPI). Non-overlapping neurites were imaged using a fluorescence microscope (Axio Observer Z1 equipped with the ApoTome.2 extension and a C-Apochromat 63x/1,2 W Korr UV-VIS-IR objective, all Zeiss) at 0.102 μm/pixel. Images were high-pass filtered using ImageJ (FFT band-pass, 0 – 15 pixels, autoscaled) and analyzed in ImageJ using a Puncta Analyzer plugin.^62^ Regions of interest for analysis were selected manually. Synaptophysin: green channel, homer1: red channel.

#### Single-nucleus RNA-sequencing

##### Isolation of nuclei for single-nucleus RNA-sequencing (snRNA-seq)

mPFC tissue from 20 weeks old male *Taok2* cKO and control mice was used for nuclei isolation. All solutions were freshly prepared and kept on ice throughout the process. The tissue samples were first incubated on ice with a 2 mL Dounce homogenizer set (Kimble Kontes, DWK Life Sciences) includes Pestle A with a large clearance (0.0030–0.0050 inch) and Pestle B with a small clearance (0.0005–0.0025 inch) in 500 μl HB buffer (0.25 M sucrose, 20 mM tricine, 25 mM KCl, 5 mM MgCl_2_, 0.5 mM spermidine , 0.15 mM spermine, 1 mM dithiothreitol (DTT), 1 mM EDTA) with 0.2 U/μl RNase inhibitor (Protector, Roche, # 3335399001) for 10 min, followed by the addition of 30 μl of 5 % IGEPAL CA-630. The tissue was then homogenized in the Dounce homogenizer while avoiding bubbles. The homogenates were transferred to 1.5 ml low binding tubes (Eppendorf, #30108051) and centrifuged at 1,000 ×g for 10 min at 4°C. The crude nuclear pellets were resuspended in 500 μl HB buffer with RNase inhibitor, followed by the addition of 30 μl of 5 % IGEPAL CA-630 and 500 μl of Working Solution C (3 ml OptiPrep (StemCell Technology) + 600 μl Solution B (500 mM tricine, 1M KCl, 1 M MgCl_2_)), and were then filtered through pre-wet 40 μm cell strainers (pluriSelect Life Science, #43-10040-40). Continuous iodixanol gradients were prepared by gently underlaying 850 μl of 30 % Gradient Solution (1.5 ml Working Solution C + 500 μl HB buffer with RNAse inhibitor + 500 μl HB buffer) and centrifugation at 10,000 × g for 25 min at 4°C. The nuclear pellets were resuspended in 100 μl of Minute Anti-Clumping Nuclei Storage Buffer (Invent Biotechnologies, #WA-014) containing 0.5 μl of RNase inhibitor. If aggregates were observed, a second filtration was performed by diluting the nuclei in PBS with 2 % BSA (Biomol, #01400) and passing the mixture through a pre-wet 40 μm cell strainer. Nuclei were counted by staining with Hoechst (Sigma-Aldrich, #94403-1ML) (0.5 μg/ml final concentration in 2 % BSA/PBS) using a Neubauer chamber (Kisker Biotech) and an EVOS microscope (Thermo Fisher Scientific), analyzing the images in ImageJ.

Single-nucleus libraries were prepared according to manufacturer protocols (Illumina, NovaSeq 6000 S1 Reagent Kit v1.5 (100 cycles)) and the Chromium Controller iX (10x Genomics). Libraries for sequencing were prepared following the manufacturer’s recommendations (Chromium NextGEM Single Cell 3’ v3.1 (10x Genomics). Average fragment size, quality, and yield of the final libraries were measured on a Bioanalyzer 2100 (Agilent). Libraries were sequenced at a final loading concentration of 300 pM (1.5 nM pool concentration) on a NovaSeq6000 instrument (Illumina) in paired-end mode.

##### snRNA-seq data analysis

BCL files were demultiplexed with bcl2fastq (Illumina) and the sequence quality was confirmed with FastQC (v0.11.9). Barcode tagging, mapping, and cell calling for 10x Genomics Next GEM libraries were done with CellRanger (v7.2; 10xGenomics). Ensembl annotation of the GRCm38 mouse genome was pre-filtered for protein-coding genes and used as mapping reference. Downstream analysis of single nucleus RNA-seq data.^68^ The count matrix filtered for CellRanger-called cells was imported into R and analyzed using Seurat v3.^69^ Cells were filtered using thresholds of *nFeature_RNA > 200* and *nFeature_RNA < 3500*. The filtered count data was normalized using LogNormalize method, regressing out mitochondrial content and cell cycle scores.^69^ Through PCA dimensionality reduction, high-dimensional data were converted into a low-dimensional representation, removing redundant information and retaining the most important features. The first 15 principal components were kept for downstream analysis. Cells were clustered using a graph-based shared nearest neighbor (SNN) clustering algorithm. Results were retrieved at a clustering resolution of 0.5 using the *FindNeighbors* and *FindClusters* functions.^70^ UMAP reduction was used for visualization. Cell cluster identities were annotated based on selective cell type markers.^31^^,^^32^ Differential expression analysis was performed using the FindMarkers function in Seurat. The Wilcoxon rank-sum test was used to identify DEGs between conditions. Genes were considered significant if they met the threshold of adjusted p-value < 0.05 (Wilcoxon rank-sum test) and absolute log2 fold change > 0.25.

##### Pathway analysis

Gene Set Enrichment Analysis (GSEA) and Over-Representation Analysis (ORA) were performed using the *clusterProfiler* R packages to identify enriched biological pathways and functional categories. For GSEA, the input was a ranked list of genes based on log2 fold change from differential expression analysis, as described in the snRNA-seq section. For ORA, DEGs with a log2 fold change threshold of 0.25 and a p-value/adjusted p-value threshold of 0.05 were used as input. To control for potential bias in gene detectability and testing, the reference gene set was defined as all DEGs identified in the dataset. Enrichment was calculated using a hypergeometric test and the Benjamini-Hochberg method was used for multiple testing correction. GO terms for biological processes (BP), cellular components (CC), and molecular function (MF), as well as KEGG pathway annotations were used for ORA and GSEA analyses. In addition, SynGO annotations were used for ORA analysis^71^ using the web-interface https://syngoportal.org/. The results were visualized using dot plots, bar plots, and network diagrams generated with the *enrichplot* and *ggplot2* packages.

For comparison with DEGs from previously published bulk RNA-seq analysis of cortices from complete *Taok2* knockout mice,^38^ the cut-off was set to an absolute log2 fold change > 0.25 and an adjusted p-value ≤0.05. Gene symbols were standardized across datasets to ensure consistency. Upregulated and downregulated genes were compared separately for GO term enrichment using ORA analysis. The overlap between DEGs from the two datasets was quantified and a Venn diagram was generated using the *VennDiagram* package in R to visualize the number of unique and shared genes.

#### Protein-protein interaction network analysis

Protein-protein interaction networks were constructed using the STRING database (https://string-db.org/). The minimum required interaction score was set to 0.4 (medium confidence). The line thickness in the network represents the strength of data support for each interaction. The degree of a node was defined as the number of edges connected to it, while the edge width was determined based on a combined score integrating data from physical interactions and predicted interactions. The resulting network was visualized using the *igraph* and *dplyr* packages in R. All significant DEGs were selected for this analysis; genes without connections were removed from the plot.

#### BioGRID interaction analysis

Physical interaction data were downloaded from the BioGRID database. The list of physical interactors was compared against our set of significant differentially expressed genes (DEGs). Genes present in both lists were identified as overlapping candidates for further analysis. ORA and visualization of the overlap were performed in R using the *clusterProfiler* R and the *VennDiagram* packages.

#### Gene-disease association analysis using the DisGeNET database

The DisGeNET database (www.disgenet.com) was queried for *Taok2*-regulated DEGs that had reached significance and results were filtered for the disease class term “mental disorders”. Gene-disease association (GDA) scores range from 0 to 1, with 1 exhibiting the highest confidence. Genes with a GDA score > 0.5 were used for plotting. GDA scores of downregulated DEGs were plotted in blue, GDA scores of upregulated DEGs were plotted in red.

#### Mouse behavior

Mouse behavior was analyzed using the PsyCoP platform.^41^ The test battery consisted of: open field test, Y-maze test, place learning, reversal learning, pre-pulse inhibition (PPI), fear conditioning, and tail suspension test. After weaning and genotyping, 23 control and 29 *Taok2* cKO mice were assigned to their final groups of ∼12 animals of both genotypes that were housed in 2000P cages (Tecniplast, 612 × 435 × 216 mm). In addition, the influence of sex on behavior was assessed, with cohort sizes for females (control, n = 15; *Taok2* cKO, n = 13) and males (control, n = 8; *Taok2* cKO, n = 16). Animals were implanted with an RFID transponder for the IntelliCages one week prior to behavioral testing. Boxplots with single data points were generated using the *ggplot2* package in R. P values were calculated using the Wilcoxon rank-sum test.

#### Behavior tests

The open field, Y-maze, tail suspension, and fear conditioning tests were recorded and analyzed in ANY-maze (Stoelting) using video cameras (DMK 22AU03, The Imaging Source). Place learning, reversal learning, circadian activity, and sucrose preference were tested in automated IntelliCage systems (TSE Systems) that consist of four automated corners for 2000P cages, each with an RFID reader and two doors with light gates that open on nosepokes and give access to water from drinking bottles. Visits to corners, nosepokes, door openings, and nipple licks were recorded.

##### Open field test

Locomotor activity, exploratory behavior, and anxiety-related behavior were analyzed in the open field test. The anxiety-related thigmotactic behavior of staying in close contact with the walls was emphasized by performing the test under regular room light at ∼1600 lx^42^^,^^72^ The arenas consisted of white rigid PVC boxes (50 × 50 × 50 cm). Animals were acclimatized to the testing room in their open home cages and behavior was recorded for 10 min. The following zones were defined for analysis: three concentric strips of 5 cm (the outer one considered as the periphery,^73^ a center of 20 × 20 cm, and four corners of 10 × 10 cm. Zone entry was analyzed based on the center of the body. For each zone, the time spent within, as a percentage of total testing time, distance travelled, and number of entries were analyzed.

##### Y-maze

The Y-maze consisted of three identical gray rigid PVC arms (inner dimensions (L×W×H): 43 × 7.5 × 10 cm), positioned at 120° angles to each other. The tests were performed at ∼15 lx. The animal was placed in the center and allowed to freely explore for 10 min. The threshold for entering an arm was 85 % of the body area and 75 % for exiting. The number of all possible three consecutive arm visits (triads) was analyzed and spontaneous alternations were calculated as the percentage of triads without change in direction relative to all visits − 2.

##### IntelliCage

IntelliCage experiments consisted of a habituation phase (2 days, all doors open; 1 day, doors open on corner entry; 2 days, doors open on nosepoke; 1 day, doors open with a 50 % chance; 1 day, doors open with a 30 % chance to facilitate visits of all four corners). For place learning (3 days), mice were pseudo-randomly assigned to one corner. A visit was counted after at least one nosepoke. A preference score was calculated for all visits in this phase. In the serial reversal test (3 days), this corner was switched daily. Learning speed in this phase was calculated by cumulative SPRT analysis as the time to reach the learning criterion of 10 % above random chance at a significance level of 5 %. For the sucrose preference test, all corners were equipped with one bottle of plain water and one bottle of a 4 % sucrose solution each. A preference score was calculated for all licks in this phase. Nocturnality was analyzed as a preference score for the first five days of habituation for corner visits during day versus nighttime. All weighted preference scores from −1 to 1 were calculated using the formula^41^

(choicesA×probabilityA−choicesB×probabilityB)÷(choicesA×probabilityA+choicesB×probabilityB).

##### Pre-pulse inhibition

Pre-pulse inhibition was tested in an SR-Lab startle response system (San Diego Instruments). White noise of 65 dBA was used as a continuous background. Mice were habituated to the enclosures for 10 min daily for three days. For the actual test, mice were habituated for 5 min followed by ten 40 ms 115 dbA pulses of white noise. Pre-pulse inhibition was tested with 20 ms pre-pulses of 70, 75, 80, or 85 dBA, a 100 ms inter-pulse interval, and a 40 ms startle pulse of 105 dBA. Additionally, 10 trials each of startle pulse-only and pre-pulse-only were included. All trials were presented in a pseudorandomized order with inter-trial intervals of 8 – 22 s.

##### Tail suspension test

For the tail suspension test, a plastic tube was placed over the tails to prevent the animals climbing their own tails. Mice attached with adhesive tape by their tail to a bar that was placed on a 30 cm high white rigid PVC rack with section dividers that was open to the front. Mice were suspended for 6 min at 1600 lx (regular room light). Time mobile and immobile, based on movement of the center of the body, the number of mobile and immobile episodes, and the latency to the first immobile phase was analyzed.^74^

##### Fear conditioning

The fear conditioning system (Ugo Basile) consisted of boxes containing a dim light, a speak, a ceiling camera, and an enclosure with a metal grid floor for delivering electrical foot shocks (17 × 17 × 25 cm), connected to a control unit. The outside walls of the enclosures were decorated with 1.5 cm wide black and white stripes. On day 1 (conditioning), baseline freezing behavior in a novel environment was recorded for two minutes. For conditioning, an auditory cue (10 kHz, 75 dBA, 20 s) was presented followed by a foot shock (2 s, 10 Hz pulses of 10 ms, 0.6 mA) and a pause of 30 s. This sequence was repeated once. On day 2 (contextual memory test), animals were placed in the same enclosure for 2 min without further intervention and freezing behavior was recorded. On day 3 (cued memory test), animals were placed in an alternative enclosure (clear plastic pipe (25 cm diam.) on a gray floor). After 2 min habituation time, the auditory cue sequence was repeated as on day 1 for 2 min and freezing was recorded for another 2 min. Percent time spent freezing was analyzed.

### Quantification and statistical analysis

Statistical significance was determined using a parametric (*t* test, ANOVA) or a non-parametric test (Wilcoxon rank-sum test). A linear mixed-effects model with Type III ANOVA using the Satterthwaite method (mixed ANOVA) was used to evaluate the interaction between genotype and time (for calcium flux measurements) and the interaction between genotype and distance (for Sholl analysis). Normality of data and variance homogeneity were assessed using the Shapiro-Wilk test and the Levene test, respectively. All analyses, if not stated otherwise, were performed using R Statistical Software (v4.2.2; R Core Team 2021). Data are presented as means +/− SD as indicated, with n = 4 for Western blots and pathwayProfiler assays, n = 6 for luciferase assays (for all these, n represents number of wells), n = 30 for calcium flux assays (n represents number of cells), n ≥ 14 for morphological analyses (n represents number of neurites/soma/intersections/puncta), and n ≥ 19 for behavioral analyses (n represents number of mice, with n = 23 for control (only n = 19 in open field test) and n = 29 for *Taok2* cKO mice). For pathway profiling assays, a *Wald* test was used to calculate an adjusted p-value with a Benjamini-Hochberg (BH) correction. For analyses of Western blots, Wilcoxon rank-sum test was applied. Significance levels indicated by asterisks are ∗ p≤0.05, ∗∗ p≤0.01, ∗∗∗ p≤ 0.001, and ∗∗∗∗ p≤ 0.0001.

## Supplemental information

Document S1. Figures S1–S11 and Tables S8 and S9

Table S1. Plasmids of the barcoded pathwayProfiler assay, related to Figures 1 and S2

Table S2. Cell-type markers for snRNA-seq analysis, related to Figures 4 and S4

Table S3. Significant DEGs from snRNA-seq analysis, related to Figures 4, S4, and S5

Table S4. KEGG pathways enriched in excitatory neurons of cortical layers 2/3 and 4/5, related to Figures 4 and S5

Table S5. GSEA based on GO term “Biological Process” (GO-BP) for excitatory neurons of cortical layers 2/3 and 4/5 and inhibitory interneurons of Sv2c type, related to Figure S6

Table S6. BioGRID interactors for human and mouse TAOK2, related to Figure S6

Table S7. Comparison of DEGs from bulk RNA-seq and snRNA-seq, related to Figure S7
