# Supplementary material for: TAOK2 controls synaptic plasticity and anxiety via ERK and calcium signaling
Source: iScience. 2025 Oct 9;28(11):113712. doi: 10.1016/j.isci.2025.113712 (PMC12590003; doi:10.1016/j.isci.2025.113712)
Supplement: Document S1. Figures S1–S11 and Tables S8 and S9 [file mmc1.pdf]

iScience, Volume *28*

Supplemental information

TAOK2 controls synaptic plasticity and anxiety via ERK and calcium signaling

Wenbo Ma, Inanna Warnhoff, Marius Stephan, Xiao Ma, Kerstin Dehne, Paul Volkmann, Nirmal Kannaiyan, Ben Brankatschk, Niels Jensen, Moritz J. Rossner, Volker Scheuss, and Michael C. Wehr

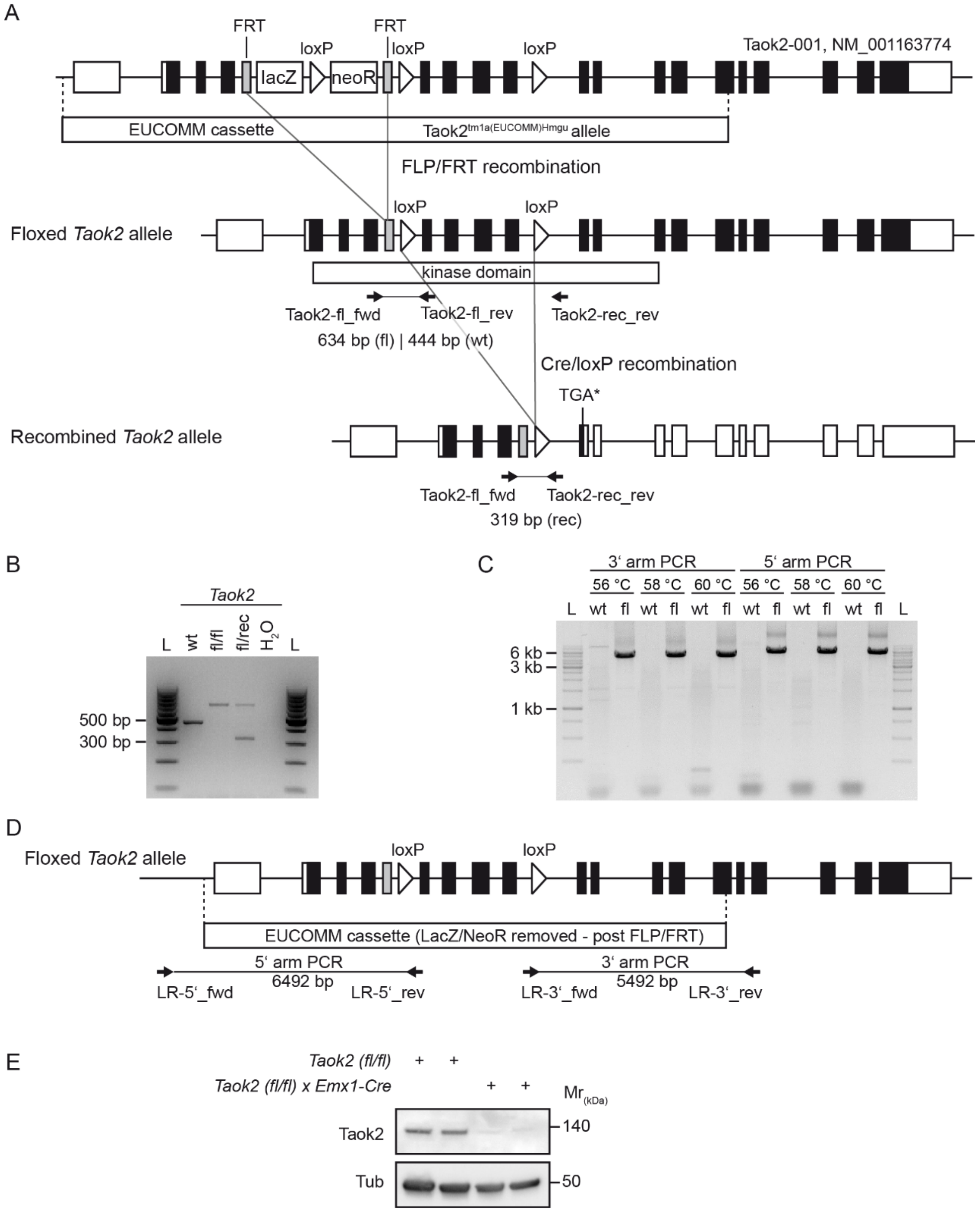

Figure S1. Generation of conditional *Taok2* cKO mice. (**A**) Schematic organization of the floxed genomic locus of mouse *Taok2*. PCR products for the floxed (*fl*), wild type (wt), and recombined (*rec*) locus are indicated. (**B**) Genotyping of wild type, floxed (*fl/fl*), and partially recombined (*fl/rec*) *Taok2* mice. L: 100 bp ladder. (**C**) Long-range PCRs upstream (5’ arm PCR) and downstream (3’ arm PCR) used to validate the correct integration of the EUCOMM transfer plasmid to introduce the floxed *Taok2* allele. (**D**) Organization of the long-range PCRs for the floxed mouse *Taok2* allele. (**E**) Western blot of prefrontal cortex lysates from of 18-weeks old control (*Taok2 (fl/fl)*) and homozygous *Taok2* cKO (*Taok2 (fl/fl) x Emx1-Cre*) mice. Taok2 protein is selectively knocked out in excitatory neurons, but not in other neural cell types.

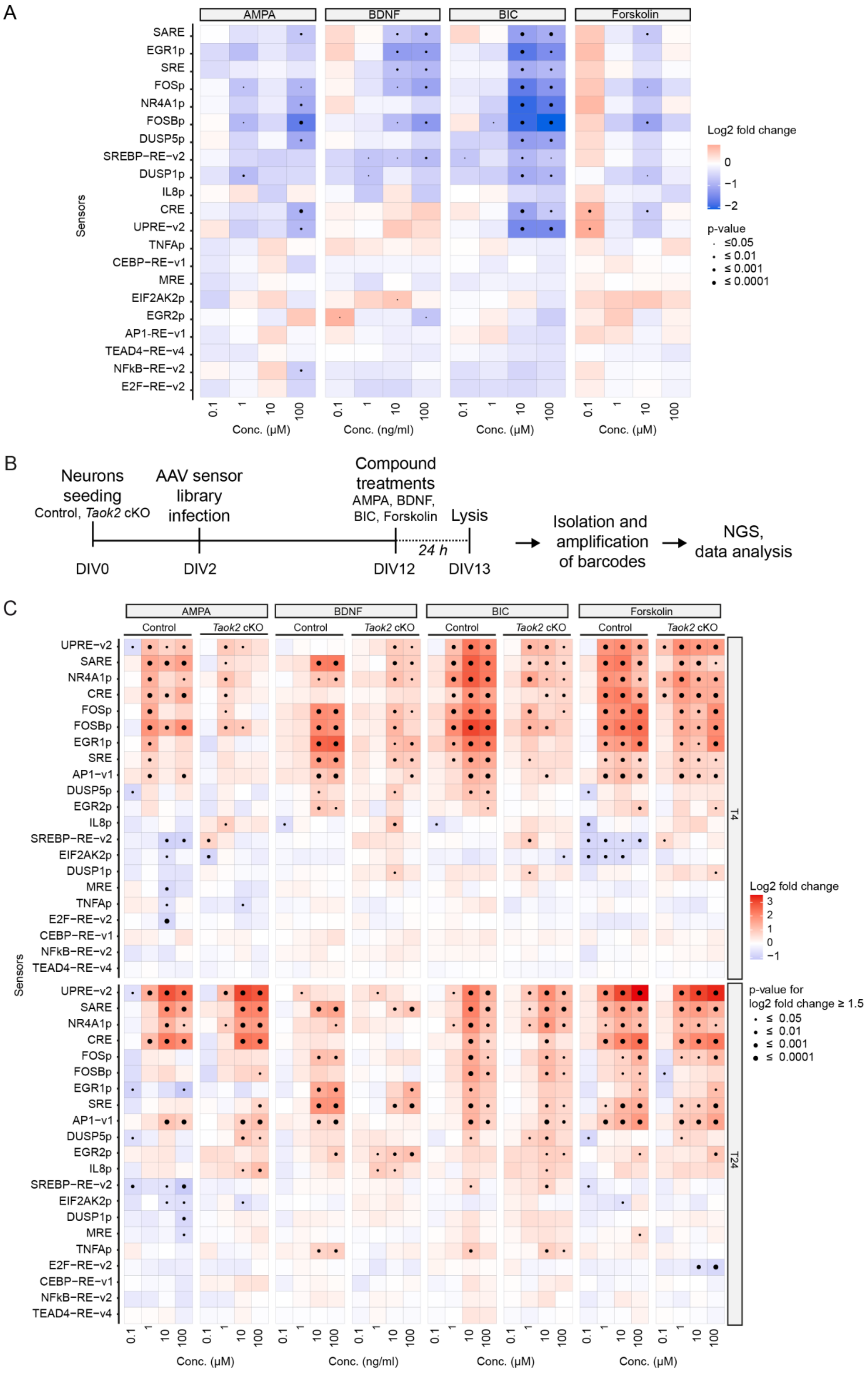
A
AMPA
BDNF
BIC
Forskolin
Sensors
SARE
EGR1p
SRE
FOSp
NR4A1p
FOSBp
DUSP5p
SREBP−RE−v2
DUSP1p
IL8p
CRE
UPRE−v2
TNFAp
CEBP−RE−v1
MRE
EIF2AK2p
EGR2p
AP1−RE−v1
TEAD4−RE−v4
NFkB−RE−v2
E2F−RE−v2
0.1
1
10
100
Conc. (µM)
Conc. (ng/ml)
Log2 fold change
0
−1
−2
p-value
≤0.05
≤ 0.01
≤ 0.001
≤ 0.0001
B
Neurons seeding
Control, Taok2 cKO
AAV sensor library infection
Compound treatments
AMPA, BDNF, BIC, Forskolin
Lysis
24 h
DIV0
DIV2
DIV12
DIV13
Isolation and amplification of barcodes
NGS, data analysis
C
AMPA
BDNF
BIC
Forskolin
Control
Taok2 cKO
Sensors
UPRE−v2
SARE
NR4A1p
CRE
FOSp
FOSBp
EGR1p
SRE
AP1−v1
DUSP5p
EGR2p
IL8p
SREBP−RE−v2
EIF2AK2p
DUSP1p
MRE
TNFAp
E2F−RE−v2
CEBP−RE−v1
NFkB−RE−v2
TEAD4−RE−v4
T4
T24
0.1
1
10
100
Conc. (µM)
Conc. (ng/ml)
Log2 fold change
3
2
1
0
−1
p-value for
log2 fold change ≥ 1.5
≤ 0.05
≤ 0.01
≤ 0.001
≤ 0.0001

Figure S2. Pathway profiling identified reduced MAPK and calcium signaling in *Taok2* cKO neurons, related to Figure 1.

(**A**) Heatmap showing the genetic effect of *Taok2* inactivation in AMPA, BDNF, BIC, and forskolin-treated samples 4 h after stimulation. n = 4. Related to Figure 1D. (**B**) Experimental timeline of the pathwayProfiler assay in mouse primary neurons with a 24 h stimulation. NGS, next-generation sequencing. (**C**) Heatmap of 24 h stimulation response of control and *Taok2* cKO primary cortical neurons to AMPA, BDNF, BIC, and forskolin. The activity of each of the 22 pathway sensors was referenced to the MLP control sensor in sample. n = 4. *, $p \leq 0.05$; **, $p \leqslant 0.01$; ***, $p \leqslant 0.001$; ****, $p \leqslant 0.0001$, and Wald test with Benjamini-Hochberg (BH) correction.

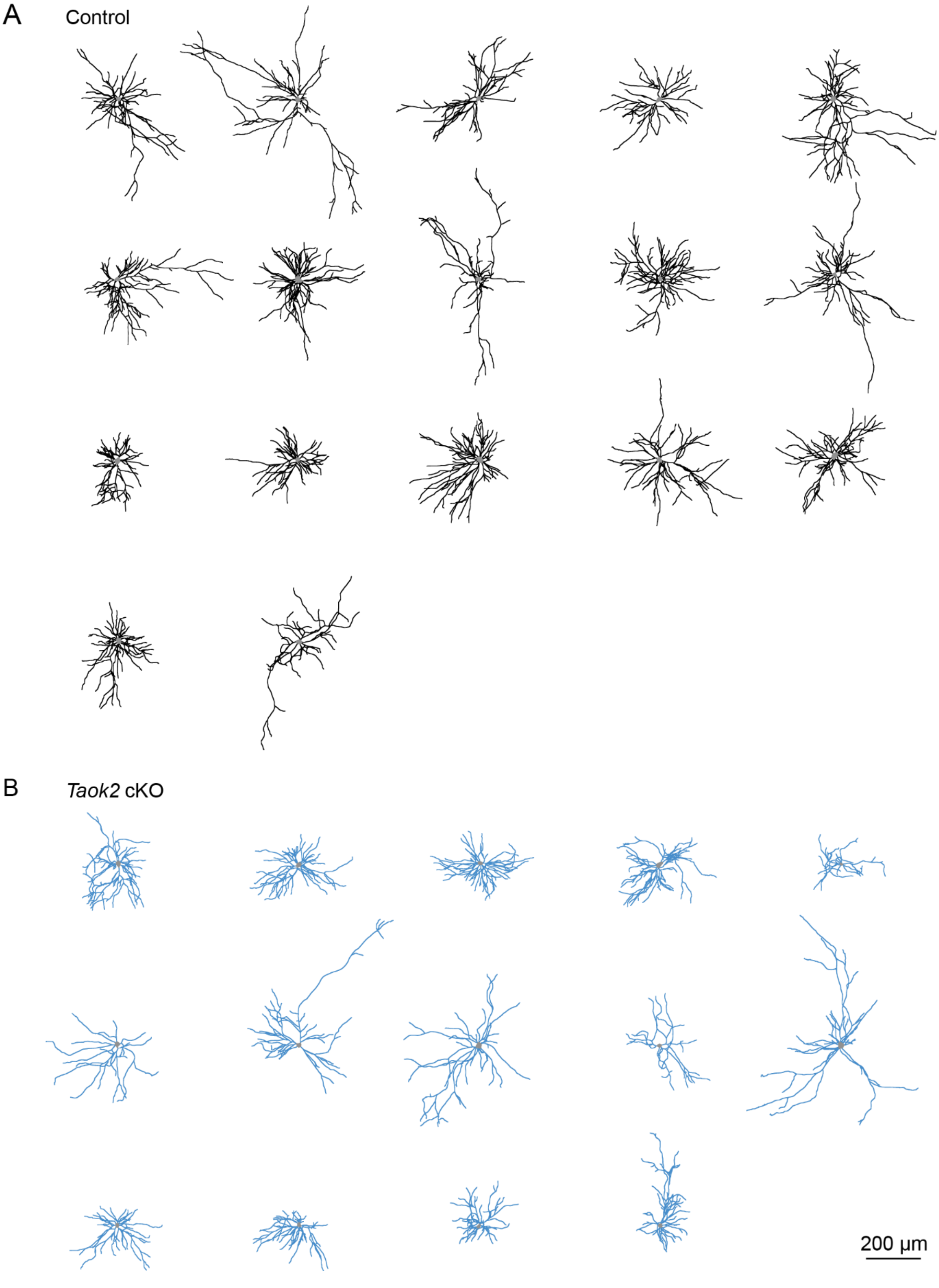

Figure S3. Representative cell morphologies used for quantification, related to Figure 3.

Neurons were imaged by 2-photon microscopy. (**A**) Control, n = 17, (**B**) *Taok2* cKO, n = 14.

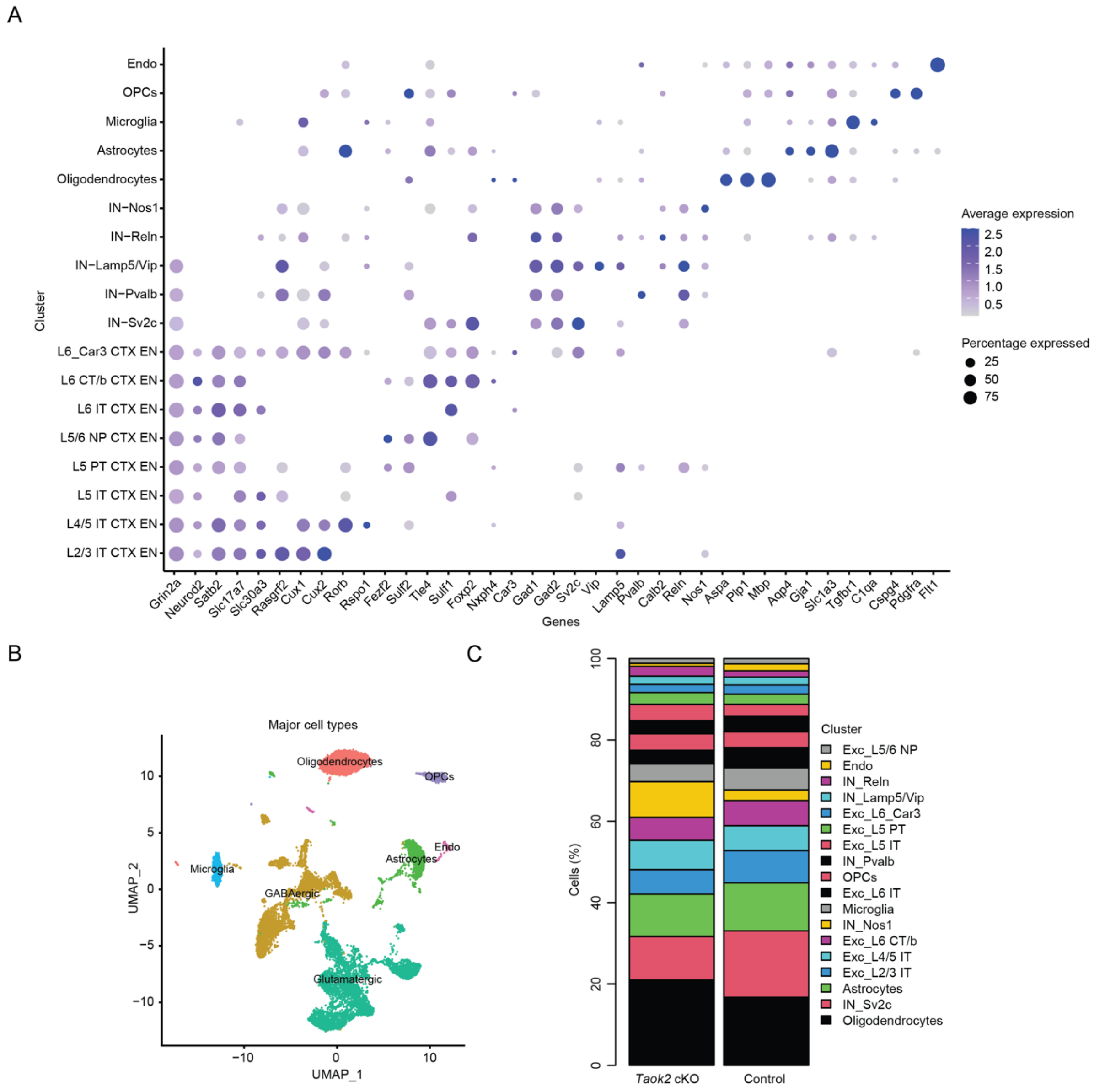

Figure S4. Identification of cell types by single-nucleus RNA-sequencing, related to Figure 4.

(**A**) Dot plot showing the expression of representative marker genes in the identified cell types. The sizes of the dots represent the percentage of nuclei expressing the marker genes and colors indicates the mean expression level. For a list of all cell type markers used, see **Table S2**. (**B**) UMAP visualization of nuclei clustered by the major cell types. (**C**) Bar plot depicting the number of nuclei per individual cell type cluster.

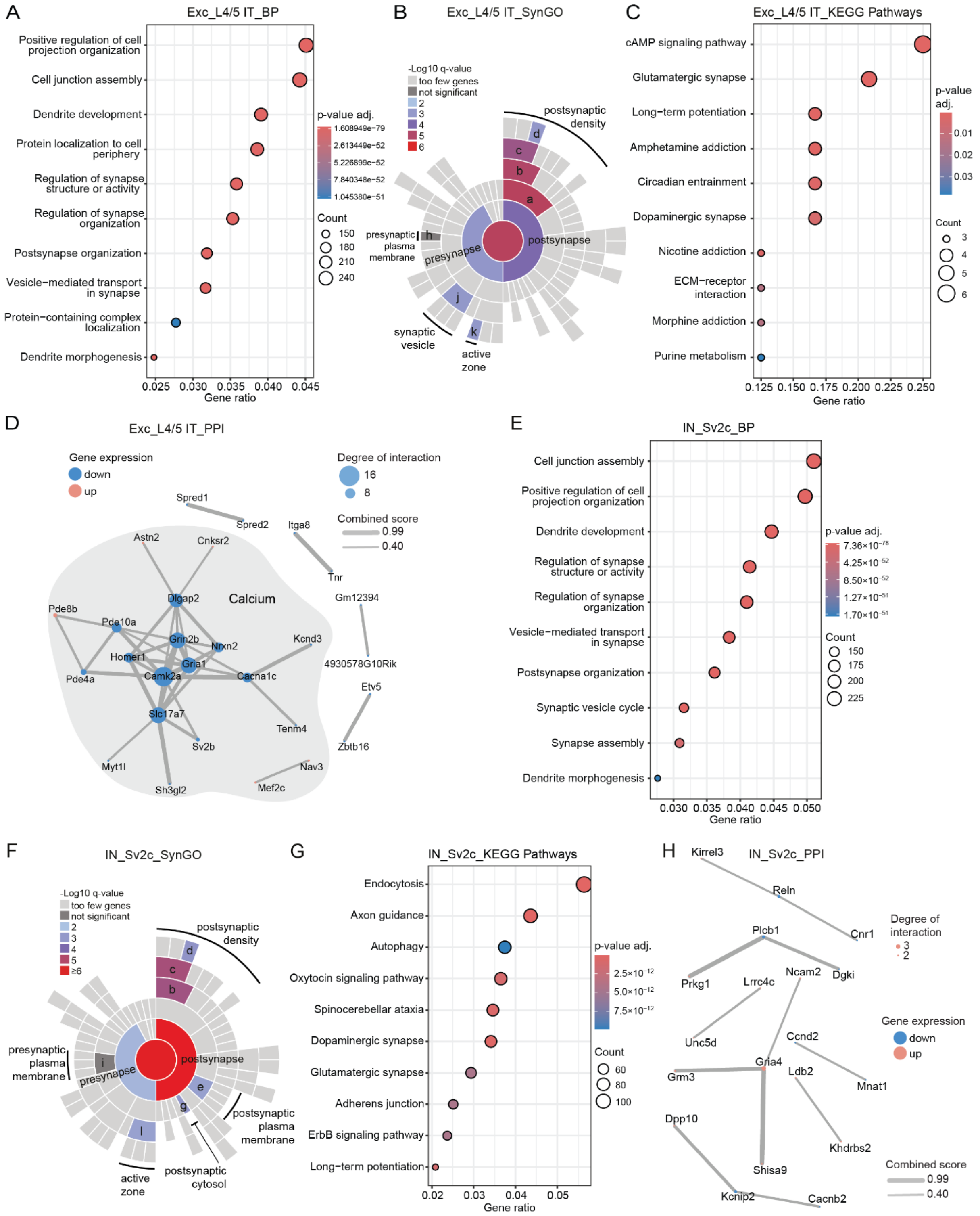

Figure S5. Enrichment analysis of biological processes and cellular components across excitatory and inhibitory neurons subtypes, related to Figure 4.

(**A-C**). Enrichment analysis for layer 4/5 excitatory neurons (Exc_L4/5 IT): (A) Dot plots of top 10 terms of GO ‘Biological Processes’ (GO-BP). (B) SynGO analysis of the significant DEGs of layer 4/5 IT excitatory neurons. SynGO analysis is based on GO ‘Cellular Components’. Genes mark each cellular state’s associated transcriptional signature. a, postsynaptic specialization; b, postsynaptic density; c, postsynaptic density membrane; d, integral component of postsynaptic density membrane; e,

postsynaptic membrane; f, integral component of postsynaptic membrane; g, postsynaptic cytosol; h, integral component of presynaptic membrane; i, presynaptic membrane; j, synaptic vesicle membrane; k, integral component of presynaptic active zone membrane; l, presynaptic active zone membrane. (C) KEGG pathways. IT, intratelencephalically projecting. For a list of all significant KEGG pathways, see **Table S4**. (**D**) Protein-protein interaction networks for Exc_L4/5 IT neurons with significant DEGs based on the STRING database. Edge width indicates the strength of data support for an interaction and is based on a combined score from experimental and predicted interactions. The minimum required interaction score was set to a medium confidence of 0.4; the maximal score was set to 0.99. The degree of a node is defined as the number of edges linking to it. (**E-G**) Enrichment analysis for interneurons (IN_Sv2c): (E) Dot plots of top 10 terms of GO-BP. (F) SynGO analysis of significant DEGs. (G) KEGG pathways. (**H**) Protein-protein interaction networks for IN_Sv2c with significant DEGs. IN_Sv2c, interneurons based on the marker Sv2c.

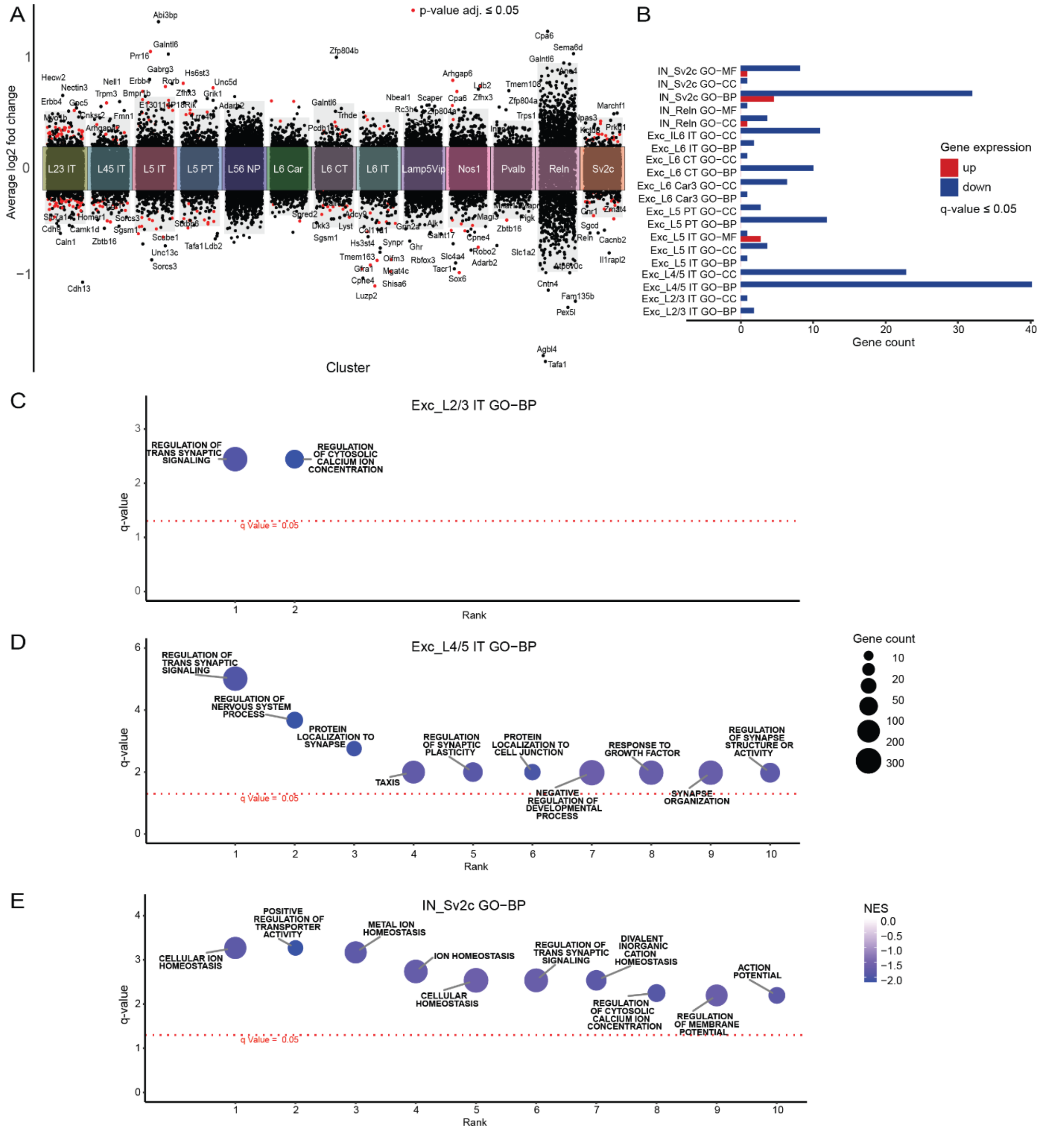

Figure S6. GSEA identified synapse related gene sets in excitatory and inhibitory neurons, related to Figure 4.

(A) Dot plot of DEGs used for GSEA and the proportion of significant DEGs (in red) in each neuron cluster. (**B**) Bar plot of significantly deregulated gene sets (q < 0.05) identified by GSEA against the GO terms ’Biological Process’ (GO-BP), ‘Cellular Component’ (GO-CC), and Molecular Function (GO-MF) collection. See **Table S5** for a list of all GO term gene sets. L2/3, layer 2/3 excitatory neurons; L4/5, layer 4/5 excitatory neurons; L5, layer 5 excitatory neurons; L6, layer 6 excitatory neurons; IN, interneurons; Car3, carbonic anhydrase 3; CT, corticothalamic; IT, intratelencephalically projecting; PT, pyramidal tract; Reln, reelin; Sv2c, synaptic vesicle glycoprotein 2C. **(C-E)** Dot plots of the ten most significantly downregulated pathways identified with GSEA based on GO-BP for (C) excitatory neurons layer 2/3, (D) excitatory neurons layer 4/5, and (E) inhibitory interneurons of Sv2c type.

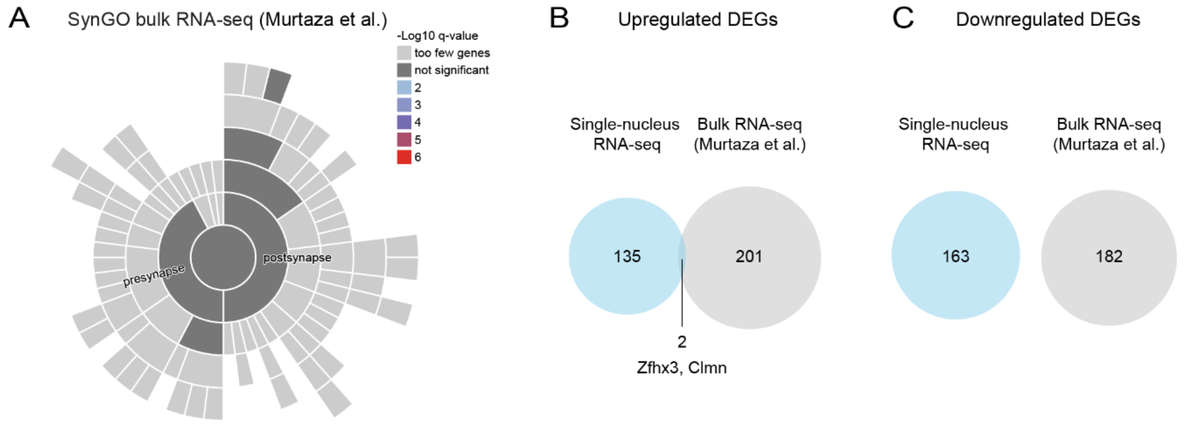

Figure S7. Comparison of single-nucleus and bulk RNA-seq datasets identified a limited overlap of TAOK2-regulated genes, related to Figure 4.

(**A**) SynGO analysis of the significant DEGs from cortices of complete *Taok2* knockout mice (Murtaza et al., 2022). SynGO analysis is based on GO 'Cellular Components'. (**B, C**) Venn diagrams showing the overlap of DEGs identified by bulk RNA-seq from Murtaza et al., 2022, and significant, unique DEGs identified by our single-nucleus RNA-seq (snRNA-seq) analysis across all neuronal cell types. (B) Two genes are shared between the upregulated DEGs identified in both data sets. (C) No overlap is observed between the downregulated DEGs from both data sets.

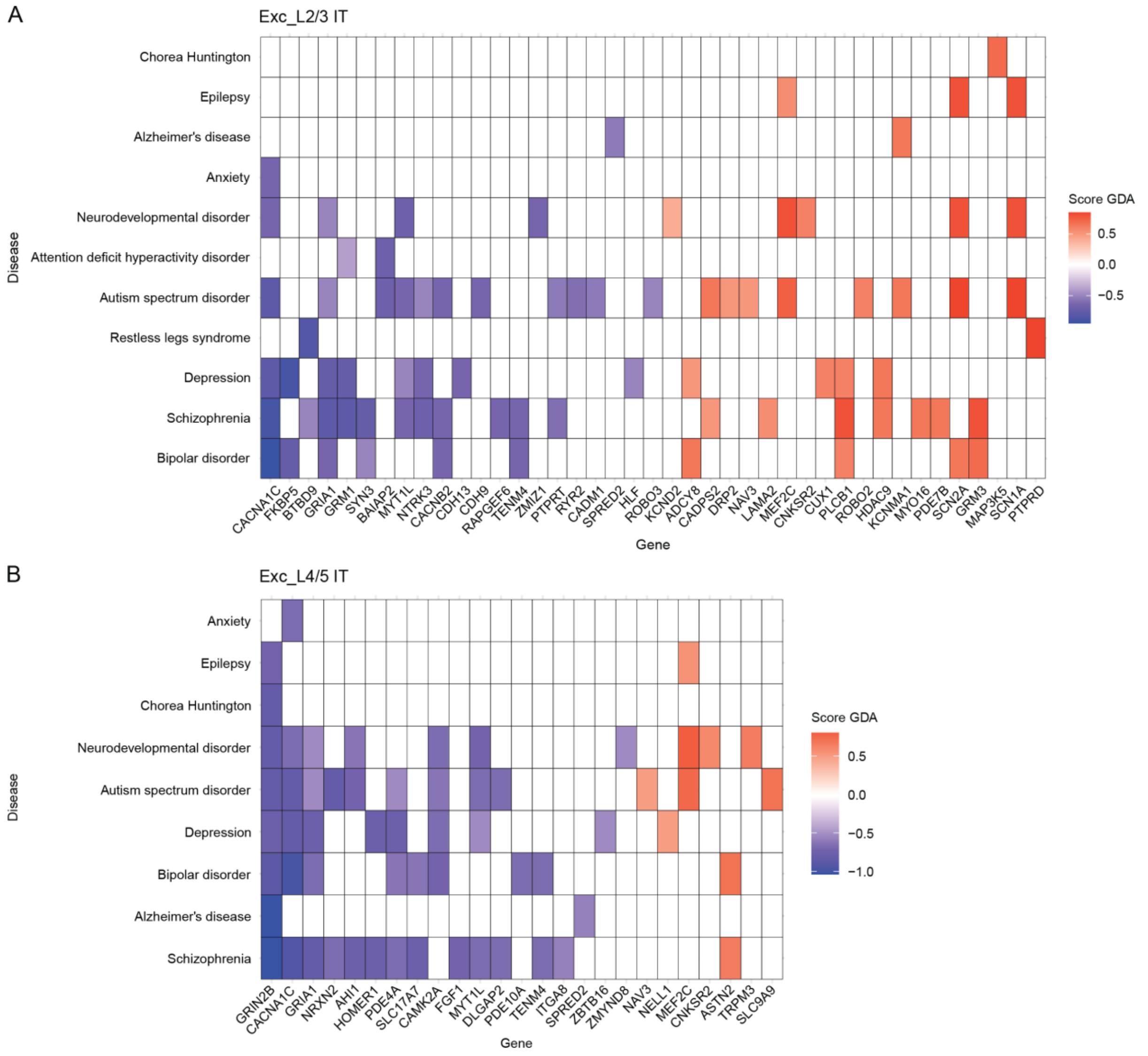

Figure S8. Gene-disease associations in L2/3 and L4/5 excitatory neurons.

(**A, B**) Heatmaps of the association between DEGs (x-axis) and mental disorders (y-axis) in (A) layer 2/3 excitatory neurons (Exc_L2/3) and (B) layer 4/5 excitatory neurons (Exc_L4/5). Rows represent disorders, including Chorea Huntington, epilepsy, Alzheimer's disease, anxiety, neurodevelopmental disorder, attention-deficit hyperactivity disorder, autism spectrum disorder, depression, restless legs syndrome, anxiety, schizophrenia, and bipolar disorder. Columns represent individual DEGs. The color scale corresponds to the gene-disease association score (Score GDA), with upregulated genes shown in shades of red and downregulated genes in shades of blue. White indicates no significant association. The cut-off for gene-disease associations shown was set to Score GDA < -0.5 (downregulated gene) and Score GDA > 0.5 (upregulated gene). IT, intratelencephalically projecting.

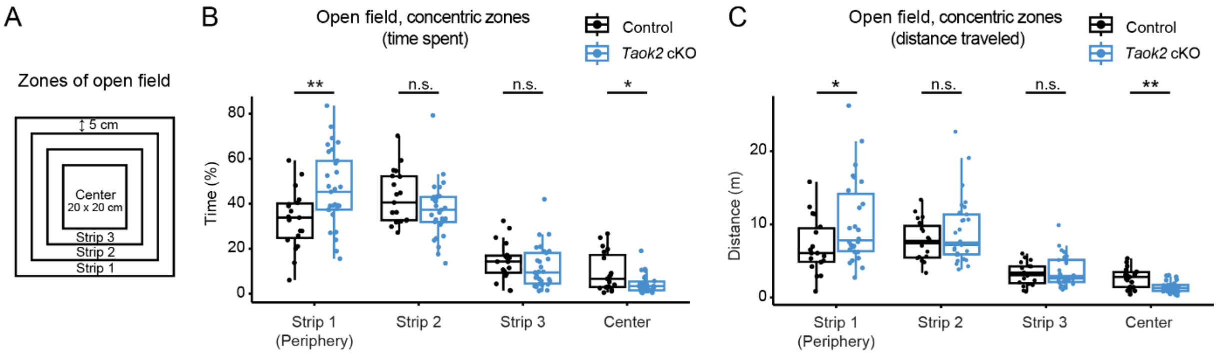

Figure S9. *Taok2* cKO mice spent more time in the corners and in direct contact with the wall in the open field test, related to Figure 5.

(**A**) Schematic of the open field arena divided into five concentric zones: strip 1 (periphery), strip 2, strip 3 (each 5 cm wide), and center (20 × 20 cm). (**B**) Time spent in each zone. *Taok2* cKO mice spent significantly more time in strip 1 in direct contact with the walls compared to controls. Strip 1, $p = 0.0084$; strip 2, $p = 0.1517$; strip 3, $p = 0.1984$; center, $p = 0.04$; with two-way ANOVA test with Tukey's post-hoc test. (**D**) Distance traveled within each zone. *Taok2* cKO mice traveled less distance in strip 1 compared to controls. Strip 1, $p = 0.038$; strip 2, $p = 0.4321$; strip 3, $p = 0.774$; center, $p = 0.0026$; with two-way ANOVA test with Tukey's post-hoc test. Cohort size: Control, $n = 19$; *Taok2* cKO, $n = 29$; *, $p \leqslant 0.05$; **, $p \leqslant 0.01$; n.s., not significant. The normality of the data sets was evaluated with the Shapiro-Wilk test.

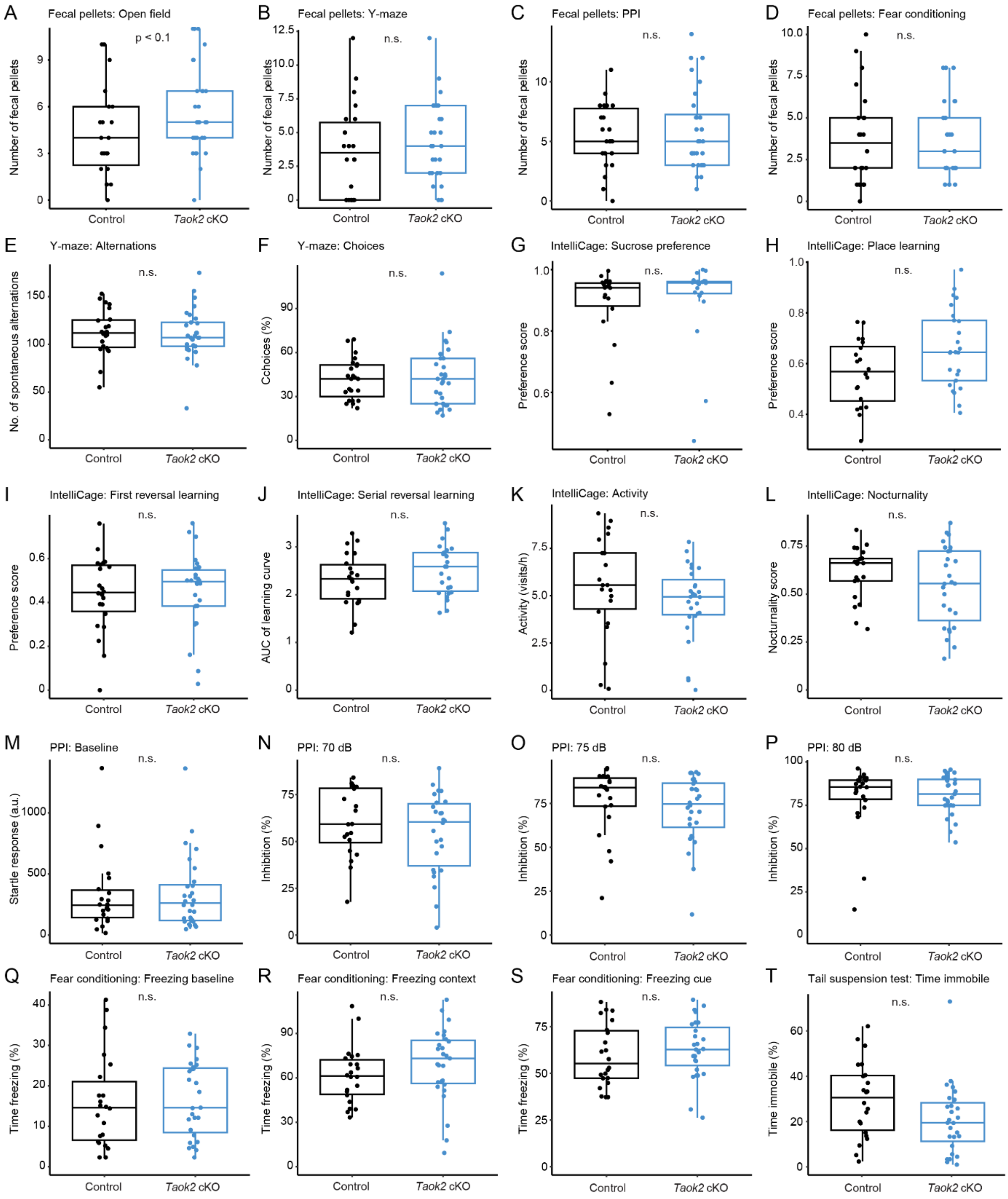

Figure S10. Behavioral assessment of *Taok2* cKO mice in tests that did not show any significant phenotypic changes, related to Figure 5.

(**A-D**) Measurement of fecal pellets between control and *Taok2* cKO mice in (A) open field, p = 0.068; (B) Y-maze, p = 0.29; (C) pre-pulse inhibition (PPI), p = 0.99, and (D) fear conditioning, p = 0.83, Wilcoxon rank-sum tests. (**E, F**) Analysis of differences between control and *Taok2* cKO mice in the Y-maze test for 10 mins for (E) choices (activity), p = 0.85, Wilcoxon rank-sum test, and (F) spontaneous alternations (working memory), p = 0.66, *t* test. (**G**) Sucrose preference, p = 0.21, Wilcoxon rank-sum test. (**H-J**) Preference score for (H) place learning, p = 0.12, *t* test; (I) first reversal

learning, p = 0.7, *t* test; and serial reversal learning, p = 0.2, Wilcoxon rank-sum test. (**K**) Activity in the IntelliCage was different in *Taok2* cKO mice. p = 0.051, Wilcoxon rank-sum test. (**L**) Nocturnality. Analysis by comparing daytime activity and night-time activity in the IntelliCage. p = 0.47, Wilcoxon rank-sum test. (**M-P**) Pre-pulse inhibition (PPI) test for sensorimotor gating: (M) baseline, (N) 70 dB, (O) 75 dB, and (P) 80 dB pre-pulse and startle pulse of 115 dB. PPI baseline, p = 0.95; 70 dB, p = 0.5; 75 dB, p = 0.2; 80 dB, p = 0.55, Wilcoxon rank-sum tests. (**Q-S**) Fear conditioning test with freezing behavior: (Q) baseline, p = 0.67; (R) cue, p = 0.35; and (S) context, p = 0.11, Wilcoxon rank-sum tests. (**T**) Time immobile in the tail suspension test, p = 0.052, Wilcoxon rank-sum test. Cohort sizes: Control, n = 23; *Taok2* cKO, n = 29; n. s., not significant. The normality of the data sets was evaluated with the Shapiro-Wilk test.

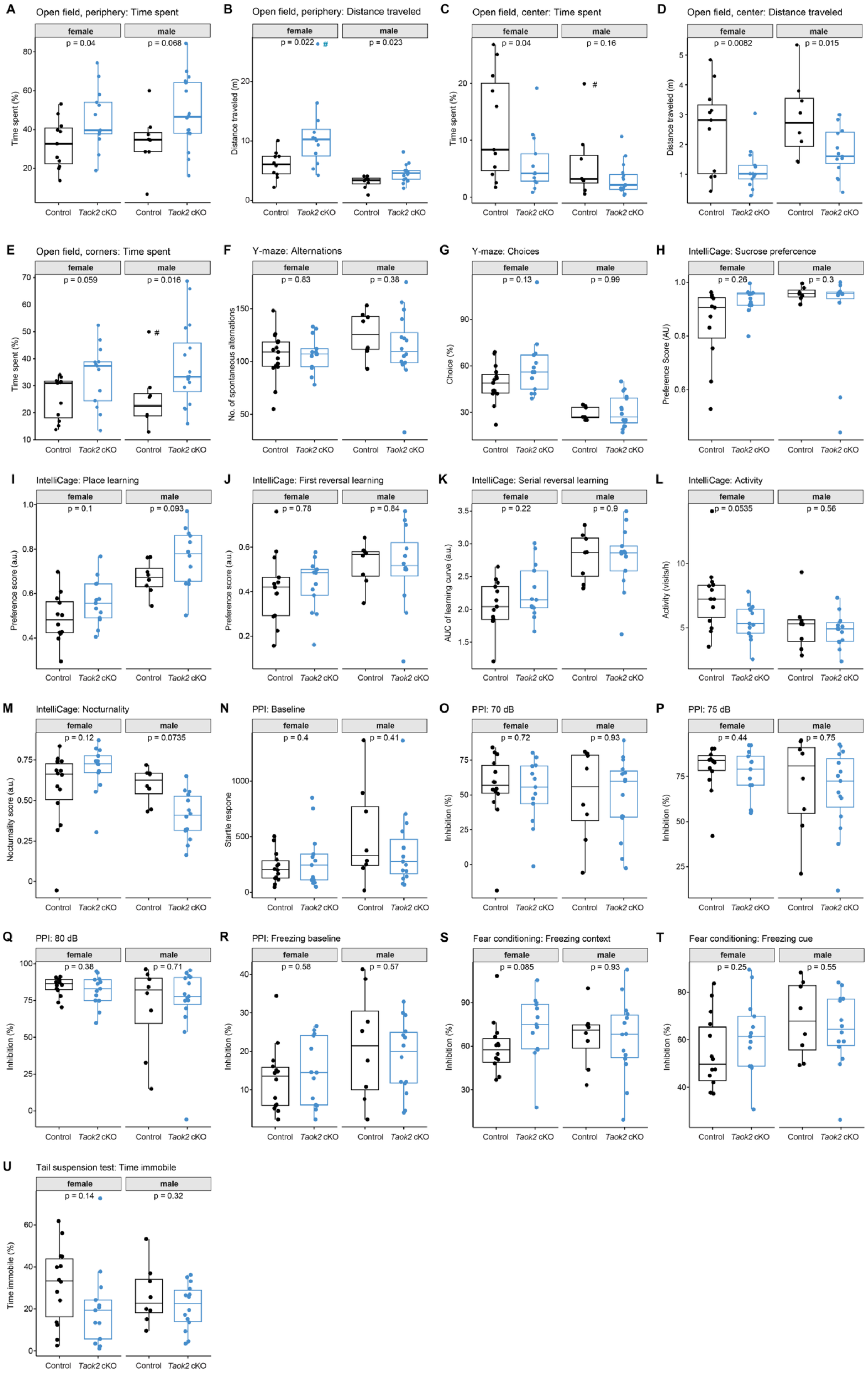
A
Open field, periphery: Time spent
female
male
p = 0.04
p = 0.068
Time spent (%)
B
Open field, periphery: Distance traveled
p = 0.022
p = 0.023
Distance traveled (m)
C
Open field, center: Time spent
p = 0.04
p = 0.16
Time spent (%)
D
Open field, center: Distance traveled
p = 0.0082
p = 0.015
Distance traveled (m)
E
Open field, corners: Time spent
p = 0.059
p = 0.016
Time spent (%)
F
Y-maze: Alternations
p = 0.83
p = 0.38
No. of spontaneous alternations
G
Y-maze: Choices
p = 0.13
p = 0.99
Choice (%)
H
IntelliCage: Sucrose preference
p = 0.26
p = 0.3
Preference Score (AU)
I
IntelliCage: Place learning
p = 0.1
p = 0.093
Preference score (a.u.)
J
IntelliCage: First reversal learning
p = 0.78
p = 0.84
Preference score (a.u.)
K
IntelliCage: Serial reversal learning
p = 0.22
p = 0.9
AUC of learning curve (a.u.)
L
IntelliCage: Activity
p = 0.0535
p = 0.56
Activity (visits/h)
M
IntelliCage: Nocturnality
p = 0.12
p = 0.0735
Nocturnality score (a.u.)
N
PPI: Baseline
p = 0.4
p = 0.41
Startle respone
O
PPI: 70 dB
p = 0.72
p = 0.93
Inhibition (%)
P
PPI: 75 dB
p = 0.44
p = 0.75
Inhibition (%)
Q
PPI: 80 dB
p = 0.38
p = 0.71
Inhibition (%)
R
PPI: Freezing baseline
p = 0.58
p = 0.57
Inhibition (%)
S
Fear conditioning: Freezing context
p = 0.085
p = 0.93
Inhibition (%)
T
Fear conditioning: Freezing cue
p = 0.25
p = 0.55
Inhibition (%)
U
Tail suspension test: Time immobile
p = 0.14
p = 0.32
Time immobile (%)
Control
Taok2 cKO

Figure S11. Genotype differences of *Taok2* cKO mice observed in the open field test remained significant when analyzing the sexes separately, related to Figure 5.

(A-C) Open field test, (A) time spent in periphery, (B) corners, and (C) center. **(D-F)** Open field test, (D) distance traveled in the periphery, (E) center, and (F) the entire arena. **(F, G)** Y-maze: (F) spontaneous alternations and (G) choices. **(H, I)** Reward-based behavior: (H) sucrose preference and (I) place learning. **(J, K)** Reversal of place learning: (J) first and (K) serial reversal learning. **(L, M)** Activity and nocturnality: (L) total activity and (M) nocturnality. **(N)** Pre-pulse inhibition (PPI) baseline startle response. **(O-Q)** PPI inhibition at (O) 70 dB, (P) 75 dB, and (Q) 80 dB. **(R-T)** Fear conditioning freezing behavior: (R) baseline, (S) context, or (T) cue. **(U)** Tail suspension test, immobility time. Cohort sizes, females: Control, n = 15 (only n = 11 in open field test); *Taok2* cKO, n = 13. Cohort sizes, males: Control, n = 8; *Taok2* cKO, n = 16. All statistical tests were run using two-way ANOVA followed by Tukey-adjusted pairwise comparisons based on estimated marginal means. #, outliers not considered for statistics.

Table S8. Oligonucleotides used for genotyping of *Taok2* cKO mice, related to Figure S1 and STAR Methods.

Oligonucleotide name	Sequence	Purpose
Taok2-fl_fwd	GACCAGTCTGGACTACCTAGTG	Genotyping Taok2 (wt/fl/rec)
Taok2-fl_rev	GAAGCTGAGCCCAGGCAATAC	Genotyping Taok2 (wt/fl/rec)
Taok2-rec_rev	CATCTTCTACTGAGGCAATGCC	Genotyping Taok2 (wt/fl/rec)
Emx1-Cre_fwd	ATTTTCCACCATATTGCCGTCT	Genotyping Emx1-Cre (tg)
Emx1-Cre_rev	AGCCATTTGACTCTTTCCACAAC	Genotyping Emx1-Cre (tg)
Emx1-wildtype_fwd	AAGGTGTGGTTCCAGAATCG	Genotyping Emx1-Cre (wt)
Emx1-wildtype_rev	CTCTCCACCAGAAGGCTGAG	Genotyping Emx1-Cre (wt)
FLIR-transgene_fwd	CACTGATATTGTAAGTAGTTTGC	Genotyping Flp (tg)
FLIR-transgene_rev	CTAGTGCGAAGTAGTGATCAGG	Genotyping Flp (tg)
FLIR-wildtype_fwd	TGTTTTGGAGGCAGGAAGCACTTG	Genotyping Flp (wt)
FLIR-wildtype_rev	AAATACTCCGAGGCGGATCACAAG	Genotyping Flp (wt)
Taok2_5arm_LR_fwd (LR-5'_fwd)	ACTATTGTGTAAGGTACTTACTGGGGATTG	Taok2 locus long-range (5')
Taok2_5arm_LR_rev (LR-5'_rev)	GGAACTTCGGAATAGGAACTTCGGTT	Taok2 locus long-range (5')
Taok2_3arm_LR_fwd (LR-3'_fwd)	GAGATGGCGCAACGCAATTAATGAT	Taok2 locus long-range (3')
Taok2_3arm_LR_rev (LR-3'_rev)	GTTCTGACACGCTAGATCACGCTC	Taok2 locus long-range (3')

Table S9. Antibodies used for Western blotting and immunocytochemistry, related to STAR Methods.

ICC, immunocytochemistry; WB, Western blot; n. a., not applicable.

Antibody	Source	Cat#	RRID identifier	WB dilution	ICC dilution
Rabbit monoclonal anti-phospho-p44/42 MAPK (Erk1/2) (Thr202/Tyr204) (clone D13.14.4E)	Cell Signaling Technology	4370	AB_2315112	1:500	n. a.
Rabbit monoclonal anti-p44/42 MAPK (Erk1/2) (clone 137F5)	Cell Signaling Technology	4695	AB_390779	1:1000	n. a.
Rabbit monoclonal anti-phospho-MEK1/2 (Ser217/221) (41G9)	Cell Signaling Technology	9154	AB_2138017	1:1000	n. a.
Rabbit monoclonal anti-MEK1/2 (D1A5)	Cell Signaling Technology	8727	AB_10829473	1:1000	n. a.
Goat polyclonal anti-TAOK2 (K-16)	Santa Cruz Biotechnology	sc-47447	AB_2240280	1:1000	n. a.
Mouse monoclonal anti-α-tubulin (clone B-5-1-2)	Sigma-Aldrich	T5168	AB_477579	1:2000	n. a.
Peroxidase-AffiniPure Goat Anti-Mouse IgG (H + L)	Jackson Immuno Research Labs	115-035-003	AB_10015289	1:2000	n. a.
Peroxidase-AffiniPure F(ab')2 Fragment Goat Anti-Rabbit IgG (H+L)	Jackson Immuno Research Labs	111-036-003	AB_2337942	1:2000	n. a.
Chicken polyclonal antibody Synaptophysin	Synaptic Systems	101006	AB_2622239	n. a.	1:500
Goat polyclonal anti-Chicken IgY (H+L) Secondary Antibody, Alexa Fluor™ 488	Thermo Scientific	A-11039	AB_2534096	n. a.	1:500
Rabbit polyclonal antibody Homer1	Synaptic Systems	160003	AB_887730	n. a.	1:500
Goat polyclonal anti-Rabbit IgG (H+L) Cross-Adsorbed Secondary Antibody, Alexa Fluor™ 647	Thermo Scientific	A-21244	AB_2535812	n. a.	1:500
